# CD8 T Cell‐Derived Exosomal miR‐186‐5p Elicits Renal Inflammation via Activating Tubular TLR7/8 Signal Axis

**DOI:** 10.1002/advs.202301492

**Published:** 2023-07-03

**Authors:** Xiaodong Xu, Shuang Qu, Changming Zhang, Mingchao Zhang, Weisong Qin, Guisheng Ren, Hao Bao, Limin Li, Ke Zen, Zhihong Liu

**Affiliations:** ^1^ National Clinical Research Center of Kidney Diseases Jinling Hospital Nanjing University School of Medicine Nanjing Jiangsu 210002 China; ^2^ School of Life Science and Technology China Pharmaceutical University 639 Longmian Avenue Nanjing Jiangsu 211198 China; ^3^ State Key Laboratory of Pharmaceutical Biotechnology Nanjing University School of Life Sciences Nanjing Jiangsu 210093 China

**Keywords:** circulating pathogenic factors, exosomal miR‐186‐5p, focal segmental glomerulosclerosis, T cells, TLR7/8

## Abstract

T cells play an important role in the development of focal segmental glomerulosclerosis (FSGS). The mechanism underlying such T cell‐based kidney disease, however, remains elusive. Here the authors report that activated CD8 T cells elicit renal inflammation and tissue injury via releasing miR‐186‐5p‐enriched exosomes. Continuing the cohort study identifying the correlation of plasma level of miR‐186‐5p with proteinuria in FSGS patients, it is demonstrated that circulating miR‐186‐5p is mainly derived from activated CD8 T cell exosomes. Renal miR‐186‐5p, which is markedly increased in FSGS patients and mice with adriamycin‐induced renal injury, is mainly delivered by CD8 T cell exosomes. Depleting miR‐186‐5p strongly attenuates adriamycin‐induced mouse renal injury. Supporting the function of exosomal miR‐186‐5p as a key circulating pathogenic factor, intravenous injection of miR‐186‐5p or miR‐186‐5p‐containing T cell exosomes results in mouse renal inflammation and tissue injury. Tracing the injected T cell exosomes shows their preferential distribution in mouse renal tubules, not glomerulus. Mechanistically, miR‐186‐5p directly activates renal tubular TLR7/8 signal and initiates tubular cell apoptosis. Mutating the TLR7‐binding sequence on miR‐186‐5p or deleting mouse TLR7 largely abolishes renal tubular injuries induced by miR‐186‐5p or adriamycin. These findings reveal a causative role of exosomal miR‐186‐5p in T cell‐mediated renal dysfunction.

## Graphical abstract

This study identifies exosomal miR‐186‐5p derived from active CD8 T cells as a circulating pathogenic factor in eliciting renal inflammation and tissue damage. CD8 T cell exosomal miR‐186‐5p is preferentially delivered into renal tubular cells where it directly binds to and activates the TLR7/8 signal pathway.

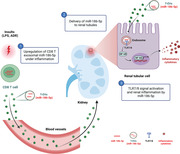

## 1 Introduction

Chronic kidney disease (CKD) is a leading case of end‐stage renal disease and is highly prevalent worldwide. As one of the leading causes of CKD, focal segmental glomerulosclerosis (FSGS) is characterized by diffuse glomerulosclerosis and tubulointerstitial in renal tissue. Although various factors, including immunologic abnormalities, genetic mutations, and certain toxins, contribute to FSGS development and progression, the mechanisms underlying FSGS pathogenesis remain incompletely understood.

It has been widely accepted that abnormal T cell activity plays an essential role in the development and progression of FSGS. Since idiopathic nephrotic syndrome (FSGS and minimal change disease) was linked to T cell dysfunction nearly half‐century ago,^[^ ^1^ ^]^ numerous studies have shown that various subsets of T cells, including CD4 T cell,^[^ ^2^, ^3^, ^4^ ^]^ CD8 T cell,^[^ ^5^ ^]^ or regulatory T cells (Tregs),^[^ ^6^, ^7^ ^]^ correlate positively or negatively with the progression of FSGS, respectively. In the doxorubicin‐induced renal injury mouse model, CD3 T cells were increased in peripheral blood and renal tissue.^[^ ^8^ ^]^ Clinical studies also found the association of CD4 T cells with nephrotic syndrome in children.^[^ ^9^, ^10^ ^]^ However, which and how T cells modulate the pathogenesis of FSGS are still unclear. Although previous studies by us^[^ ^11^ ^]^ and others^[^ ^12^ ^]^ demonstrated that podocytes particularly under inflammatory conditions could behave as antigen‐presenting cells to activate specific T cells, which in turn, attacked podocytes, renal histopathology analysis failed to find significant T cell infiltration in glomeruli of FSGS patients.^[^ ^13^ ^]^ Lack of T cell infiltration in glomeruli of FSGS patients suggests that, instead of directly attacking intrinsic renal cells, T cells may release certain soluble factors, including inflammatory cytokines, to cause renal dysfunction.^[^ ^1^, ^14^, ^15^ ^]^

Cell‐secreted exosomes are novel mediators of cell‐to‐cell communication.^[^ ^16^ ^]^ Accumulating evidence indicates that exosomes contain various miRNAs and can deliver them to the recipient cells where they modulate the expression level of the target genes.^[^ ^17^, ^18^ ^]^ Roles of extracellular vesicles (EVs) released by immune cells have been widely observed in various diseases. For instance, Salvia et al. reported that T cell‐derived EVs facilitated the pathogenesis of essential hypertension.^[^ ^19^ ^]^ Considering that T cell number and activity are markedly increased in the peripheral blood of FSGS patients,^[^ ^13^ ^]^ the exosomes released by activated T cells may contribute to renal inflammation and tissue injury in FSGS disease. Supporting the role of exosomes, particularly their miRNA content in renal function, Lv et al. reported that exosomal miR‐19b‐3p of tubular epithelial cells could cause kidney damage via promoting M1 macrophage activation.^[^ ^20^ ^]^ Dependent upon their source difference, the function of exosomes in acute kidney injury (AKI) can be different. Cao et al. showed that exosomes from mesenchymal stem cells attenuated cisplatin‐induced AKI.^[^ ^21^ ^]^ Taking advantage of the exosomal delivery system, Kim et al. reported that exosomal super‐repressor I*κ*B*α* ameliorated kidney ischemia‐reperfusion injury.^[^ ^22^ ^]^ Through retrospective and prospective cohort studies using different independent groups of FSGS patients, we previously identified a positive correlation of plasma level of miR‐186‐5p with proteinuria level in FSGS patients.^[^ ^23^ ^]^ However, whether and how circulating miR‐186‐5p causes renal dysfunction remains unknown.

In the present study, we demonstrate that circulating miR‐186‐5p is mainly derived from activated CD8 T cell‐secreted exosome, and serves as a circulating pathogenic factor driving renal inflammation and tissue injury in FSGS patients and adriamycin (ADR)‐induced renal damage mouse model. Our results further reveal that exosomal miR‐186‐5p released by activated CD8 T cells mediates renal dysfunction via activating renal tubular TLR7(murine)/8(human) signal axis.

## 2 Result

### 2.1 External Source miR‐186‐5p Initiates Renal Inflammation in ADR‐Treated Mice and FSGS Patients

**Figure 1:**
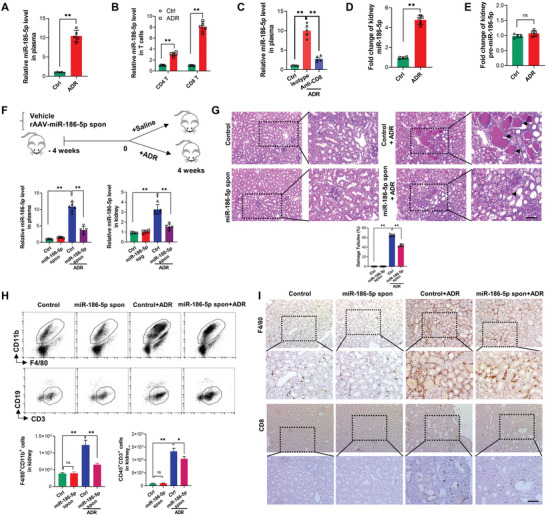
External source miR‐186‐5p initiates renal tubular inflammation and tissue injury in the ADR mouse model. A,B) ADR treatment elevated miR‐186‐5p level in A) mouse plasma particularly B) circulating CD8 T cells. C) Depletion of CD8 T cell with anti‐CD8 antibody in ADR mouse decreased miR‐186‐5p level in plasma. D,E) ADR treatment increased the level of D) miR‐186‐5p but not E) pre‐miR‐186‐5p in the mouse kidney. F) Top: Schematic of adenovirus‐based miR‐186‐5p sponge treatment. Bottom: miR‐186‐5p sponge abolished the increase of miR‐186‐5p level in mouse plasma (left) and kidney (right) by ADR treatment. G) Adenovirus‐based miR‐186‐5p sponge attenuated renal tubular injury in ADR mice. H) Flow cytometry analysis of infiltration of macrophages (F4/80^+^CD11b^+^) and T cells (CD45^+^CD3^+^) in ADR mice with or without miR‐186‐5p sponge treatment. I) Tissue staining of macrophages and CD8 T cells in mouse kidney with or without ADR plus miR‐186‐5p sponge treatment. Scale bars, 50 µm. G,I) There were five mice per group; 6–8 fields were analyzed for each mouse. A—H) Data were analyzed by unpaired two‐sided Student's *t‐*test. *, *p* < 0.05 and **, *p* < 0.01. ns, no significance.

Our previous study revealed the circulating miR‐186‐5p as a biomarker for FSGS patients.^[^ ^23^ ^]^ To further explore the role of miR‐186‐5p in renal inflammation and tissue injury in FSGS patients, we assessed the expression pattern and function of miR‐186‐5p using a renal injury mouse model induced by ADR (Figure S1A, Supporting Information). Successful establishment of the ADR‐injured mouse model was evidenced by significantly higher levels of urinary albumin and serum creatinine (Figure S1B, Supporting Information), severe glomerular sclerosis and tubular damage (Figure S1C, Supporting Information) and typical podocyte effacement (Figure S1D, Supporting Information) in ADR‐treated group compared with the group of control. Similar to the finding in FSGS patients, we detected a markedly higher level of miR‐186‐5p in plasma (**Figure** **1**A) and circulating T cells (Figure 1B) in ADR‐treated mice compared to the control group. Particularly, the miR‐186‐5p level was strikingly increased in CD8 T cells following ADR treatment. Supporting the notion that active CD8 T cells are a major source of circulating miR‐186‐5p, depletion of peripheral CD8 T cells by anti‐CD8 antibody^[^ ^24^ ^]^ (Figure S2, Supporting Information) strongly reduced plasma miR‐186‐5p level (Figure 1C). In line with this, ADR treatment significantly increased the levels of CD8 cells in mouse spleen and peripheral blood (Figure S3, Supporting Information). The selective increase of miR‐186‐5p level in CD8 T cells under inflammation was further validated using human blood samples. In this experiment, blood drawn from five healthy donors (each 2 mL) was divided into two groups, and treated with 200 ng ml^−1^ LPS or saline at 37 °C for 6 h, respectively. After the removal of red blood cells and sorting out various white blood cells, qRT‐PCR assay showed a strong elevation of miR‐186‐5p level in CD8 T cells but not other white blood cells (Figure S4, Supporting Information).

ADR mice also displayed a high level of miR‐186‐5p in kidney tissues compared to the control group (Figure 1D). To our surprise, the level of miR‐186‐5p precursor (pre‐miR‐186‐5p) in mouse kidneys did not change following ADR treatment (Figure 1E ). Similar results were observed in FSGS patient kidney tissues (Figure S5, Supporting Information). As shown, the miR‐186‐5p level in the kidney tissue of FSGS patients was significantly higher than that in control (para‐cancerous) kidney tissue, whereas pre‐miR‐186‐5p levels remained similar. These results suggest that the upregulation of renal miR‐186‐5p in ADR mice or FSGS patients is not from de novo synthesis but derived from external sources. Given that the plasma and particularly the circulating CD8 T cells contain a high level of miR‐186‐5p following ADR treatment or under FSGS conditions, the increased miR‐186‐5p level in the kidney under disease conditions may come from activated CD8 T cells.

To test whether circulating miR‐186‐5p is a pathogenic factor of kidney inflammation and tissue injury, we constructed a recombinant adenovirus‐based miR‐186‐5p sponge (spon) and employed it to decrease miR‐186‐5p level in mouse kidney (Figure 1F). After 4 weeks of miR‐186‐5p sponge administration, qRT‐PCR assay showed that ADR‐induced increase of miR‐186‐5p level in mouse plasma (Figure 1F, left) and kidney tissues (Figure 1F, right) was largely abolished. As shown in Figure 1G, depletion of kidney miR‐186‐5p by miR‐186‐5p sponge markedly attenuated renal tubular injury in ADR mice. In line with this, both flow cytometry analysis (Figure 1H) and immunohistochemical staining (Figure 1I) of immune cell infiltration indicated significantly more infiltrated F4/80^+^CD11b^+^ macrophages and CD8 T cells in the kidney in ADR‐treated mice than in non‐treated mice (Ctrl). The ADR‐induced kidney infiltration of immune cells, however, is strongly blocked by the miR‐186‐5p sponge. It has been shown that monocytes, as major inflammatory innate immune cells, are activated under renal inflammation. These results collectively suggest that external source miR‐186‐5p may play a critical role in mouse renal inflammation and tissue injury induced by ADR treatment.

**Figure 2:**
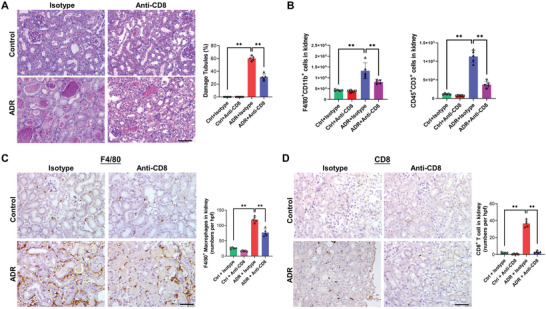
CD8 T cell depletion alleviates renal inflammation and tissue injury in the ADR mouse model. A) Depletion of CD8 T cells by CD8 antibody attenuated renal tubular injury in ADR mice. B) Flow cytometry analysis of kidney infiltration of macrophages (F4/80^+^CD11b^+^) and T cells (CD45^+^CD3^+^) in ADR mice with isotype or anti‐CD8 Ab treatment. C,D) Tissue staining of mouse kidney infiltration of C) macrophages and D) CD8 T cells with or without ADR plus isotype or anti‐CD8 Ab treatment. Scale bars, 50 µm. A,C,D) There were five mice per group; 6–8 fields were analyzed for each mouse. A—D) Data were analyzed by unpaired two‐sided Student's *t‐*test. **, *p* < 0.01.

Given active CD8 T cells as a major source of miR‐186‐5p, the depletion of CD8 T cells by anti‐CD8 mAb ^[^ ^24^ ^]^ also significantly mitigated tissue injury and renal inflammation in the ADR mouse model (**Figure** **2**). As shown, ADR‐induced renal tubular injury was markedly attenuated by CD8 cell depletion (Figure 2A). Flow cytometry analysis (Figure 2B) and immunohistochemical staining (Figure 2C,D) indicated that anti‐CD8 Ab treatment reduced the kidney infiltration of macrophages (F4/80^+^CD11b^+^) and CD8 T cells in ADR mice.

### 2.2 Renal miR‐186‐5p in ADR Mice or FSGS Patients is Mainly Derived from CD8 T Cell Exosomes

**Figure 3:**
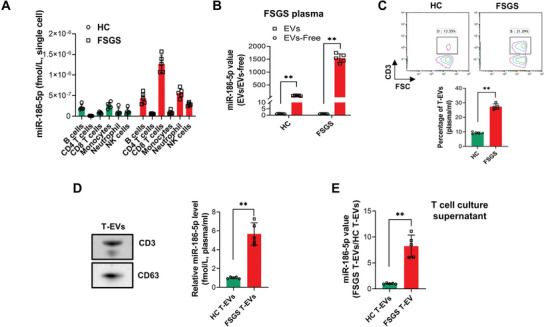
Circulating CD8 T‐EVs from FSGS patients contain higher level of miR‐186‐5p compared to that from healthy donors. A) Level of miR‐186‐5p in various peripheral leukocytes isolated from 5 FSGS patients and healthy donors. B) Distribution of plasma miR‐186‐5p from FSGS patients in EVs and EV‐free fraction. C) Level of T‐EVs in peripheral blood from FSGS patients and healthy donors (HC). D) miR‐186‐5p level in the T‐EVs from FSGS patients and healthy donors. E) miR‐186‐5p level in exosomes secreted from T cells that were isolated from FSGS patients or healthy donors. A—D) There were five patients or healthy donors per group and three independent tests from each group. In panel (E), *n* = 5. B—E) Data were analyzed by unpaired two‐sided Student's *t‐*test. **, *p* < 0.01.

To verify the notion that kidney miR‐186‐5p under renal inflammation or tissue injury is derived from circulating immune cells, particularly CD8 T cells, we compared the miR‐186‐5p expression level in various circulating leukocytes in FSGS patients and healthy donors (HC) (Table S1, Supporting Information). As shown in Supplementary Figure 6, miR‐186‐5p level in plasma was positively correlated with the number of T cells (CD3^+^) particularly CD8 T cells (CD3^+^CD8^+^) but not neutrophils, monocytes, eosinophils, basophils, and B cells. We next isolated different white blood cells (CD19 B cells, CD4 T cells, CD8 T cells, CD14 monocytes, CD16 neutrophil, CD56 NKs) in peripheral blood from FSGS patients and healthy donors and assessed the expression of miR‐186‐5p (**Figure** **3**A). The results confirmed that CD8 T cells expressed the highest level of miR‐186‐5p among all circulating leukocytes tested in FSGS patients. Supporting the role of CD8 T cells in renal inflammation and tissue injury in FSGS, the number of CD8 T cells in the peripheral blood of FSGS patients was found positively correlated with the level of proteinuria and tubulointerstitial lesions (Table S2, Supporting Information). Analysis of plasma miR‐186‐5p distribution further indicated that most circulating miR‐186‐5p in both healthy donors and FSGS patients was encapsulated in EVs (Figure 3B), suggesting that EVs mediate the transport of miR‐186‐5p from peripheral blood to the kidney. To validate this, we examined the miR‐186‐5p level in T cell‐derived exosomes (T‐EVs) in peripheral blood from healthy donors and FSGS patients. As shown in Figure 3C, flow cytometry confirmed that FSGS patients had significantly more T‐EVs than healthy donors did. The qRT‐PCR assay indicated that T‐EVs from FSGS patients contained higher miR‐186‐5p levels compared to T‐EVs from healthy donors (Figure 3D). We also isolated T cells from FSGS patients and healthy donors and monitored the secretion of miR‐186‐5p via exosomes. In this experiment, CD8 T cells isolated from FSGS patients and healthy donors were activated by anti‐CD3/CD28 antibody and rIL‐2 in the presence of 10% autologous serum. The T‐EVs were collected from the culture medium and the level of miR‐186‐5p in T‐EVs was assayed by qRT‐PCR. The result clearly showed that FSGS T‐EVs contained higher levels of miR‐186‐5p than control T‐EVs (Figure 3E).

To further explore the cellular origin of circulating miR‐186‐5p, we also established a mouse model of T lymphocyte activation using LPS intraperitoneal administration (Figure S7A, Supporting Information). The result showed that the level of urinary albumin was significantly increased on day 2 post‐LPS treatment (Figure S7B, Supporting Information). Accompanied by an increase in the number of T cell subsets of peripheral blood and spleen, miR‐186‐5p in individual peripheral CD8 T cells (Figure S7C, Supporting Information) and spleen CD8 T cells (Figure S7D, Supporting Information) was markedly upregulated following LPS treatment.

**Figure 4:**
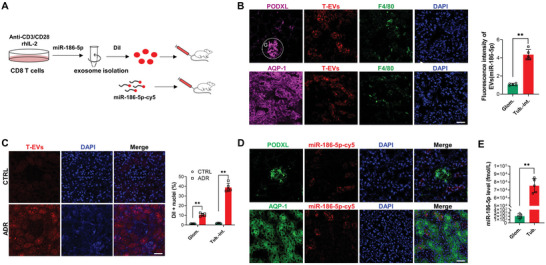
Transfer of miR‐186‐5p from circulating CD8 T cells to renal tubulointerstitium via T‐EVs. A) Schematic of T‐EVs/miR‐186‐5p tracing experiment. B) Distribution of DiI ‐labeled T‐EVs in mouse glomeruli and renal tubulointerstitium. C) ADR treatment promoted the accumulation of DiI‐labeled T‐EVs in mouse renal tubulointerstitium. D) Distribution of miR‐186‐5p in mouse glomeruli and renal tubulointerstitium following tail vein injection of miR‐186‐5p‐Cy5. E) qRT‐PCR detection of miR‐186‐5p in isolated mouse glomerulus and renal tubules 0.5 h after injection of miR‐186‐5p‐Cy5. Scale bars, 50 µm. B—E) There were five mice per group; B—D) 6–7 fields were analyzed for each mouse. Data were analyzed by unpaired two‐sided Student's *t‐*test (B–E). **, *p* < 0.01.

To test whether T‐EVs released from activated CD8 T cells can enter renal tissue, we performed a miR‐186‐5p tracing assay with different strategies. As depicted in **Figure** **4**A, mouse CD8 T cells were transfected with or without miR‐186‐5p‐Cy5 and activated by CD3/CD28 ligation. T‐EVs were then collected and labeled with or without DiI prior to tail vein injection. Firstly, we traced the DiI‐labeled CD8 T‐EVs in mouse kidneys 3 h following the injection. The results showed significant infiltration of DiI‐labeled CD8 T‐EVs in mouse kidneys (Figure 4B). Interestingly, DiI‐labeled CD8 T‐EVs were mainly accumulated in the tubulointerstitium but not the glomeruli. As expected, a certain degree of infiltration of F4/80‐positive macrophages in mouse kidneys was observed, and some macrophages were co‐localized with T‐EVs, suggesting that kidney‐infiltrated macrophages may be involved in uptaking T‐EVs and eliciting renal inflammation. Secondly, to test whether renal inflammation and tissue injury increase the accumulation of CD8 T‐EVs in the kidney, we compared the renal distribution of DiI‐labeled CD8 T‐EVs in mice treated with or without ADR for 4 weeks. As shown by ex vivo imaging of kidney tissue (Figure 4C), a significantly higher fluorescence signal was observed in the tubulointerstitium of ADR‐treated mice compared to that in mice without nephropathy. Finally, we directly traced the Cy5‐labeled miR‐186‐5p following the injection of CD8 T‐EVs. In this experiment, PODXL and AQP‐1 served as markers for glomeruli and proximal renal tubular cells (PTCs), respectively. The results confirmed the accumulation of miR‐186‐5p‐Cy5 in PTCs (Figure 4D). The qRT‐PCR analysis also showed markedly a higher level of miR‐186‐5p in mouse renal tubules than in glomeruli 30 min after injection of miR‐186‐5p‐Cy5 (Figure 4E).

### 2.3 T Cell Exosomal miR‐186‐5p Directly Activates Renal Tubular TLR7/8 Signal Axis

To validate the role of exosomal miR‐186‐5p in renal inflammation and tissue injury, we injected mice with cholesterol‐modified miR‐186‐5p anti‐sense oligonucleotides (ASO) in the LPS‐induced mouse renal injury model. In this experiment, 10 nmol of synthetic cholesterol‐modified miR‐186‐5p anti‐sense oligonucleotides (ASO) (AGCCCAAAAGGAGAATTCTTTG) or oligonucleotide control were dissolved in 150 µL of PBS with or without LPS and then injected into C57BL/6 mice twice via tail vein. Mice were euthanized 3 days following treatment with or without LPS/miR‐186‐5p ASO, and the renal injury and immune cell infiltration were assessed. As shown in Figure S8, Supporting Information, miR‐186‐5p ASO markedly mitigated LPS‐induced renal tubular injury (Figure S8A, Supporting Information). The miR‐186‐5p ASO also blocked kidney infiltration of macrophages and CD8 T cells assayed by flow cytometry (Figure S8B, Supporting Information) and immunohistochemical staining (Figure S8C, Supporting Information).

**Figure 5:**
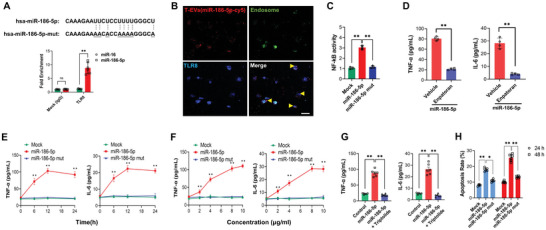
GU‐enriched miR‐186‐5p binds to endosomal TLR8 and activates TLR‐mediated inflammatory signals in HK2 cells. A) Upper: miR‐186‐5p and miR‐186‐5p‐Mut sequences; Lower: Detection of miR‐186‐5p but not miR‐16 in the anti‐TLR8 antibody‐immunoprecipitated complex by qRT‐PCR. B) Colocalization of miR‐186‐5p‐Cy5 with TLR8 in endosomal structures of human HK2 cells. C) Enhancement of NF‐*κ*B activity in HEK293T by miR‐186‐5p but not miR‐186‐5p‐Mut via activating TLR8. D) TLR8 inhibitor enpatoran (100 nM) suppressed inflammatory cytokines expression induced by miR‐186‐5p. E,F) miR‐186‐5p but not miR‐186‐5p‐Mut upregulated inflammatory cytokines express in HK2 cells in E) time and F) dose‐dependent manner. G) NF‐*κ*B inhibitor triptolide (30 nM) inhibited inflammatory cytokine expression induced by miR‐186‐5p. H) miR‐186‐5p but not miR‐186‐5p‐Mut time‐dependently promoted HK2 cell apoptosis. Scale bars, 50 µm. 3–6 independent experiments were performed in panels (A,C,D–H. Data were analyzed by A,C,D,G,H) unpaired two‐sided Student's *t*‐test or E,F) two‐way ANOVA with Dunnett's multiple comparisons test. *, *p* < 0.05 and **, *p* < 0.01. ns, no significance.

Previous studies have revealed that certain miRNAs with GU/UU‐rich sequences contributed to the innate immune response by directly binding to TLR7 (murine) or TLR8 (human).^[^ ^25^ ^]^ Considering that miR‐186‐5p also has GU/UU‐rich sequence (**Figure** **5**A, top) and circulating miR‐186‐5p is predominantly delivered to renal tubular cells which highly express TLR7/8,^[^ ^26^ ^]^ we postulate that miR‐186‐5p may activate renal tubular TLR7/8 signal axis and initiate renal inflammation. To test this hypothesis, the direct binding of miR‐186‐5p to TLR8 was examined in HK2 cells. Supporting that GU‐rich sequence mediates the miR‐186‐5p‐TLR8 binding, immunoprecipitation confirmed a specific association of TLR8 with miR‐186‐5p but not miR‐16, a miRNA without GU‐rich sequence (Figure 5A, bottom). To abolish miR‐186‐5p‐TLR7/8 binding, we also generated a miR‐186‐5p mutant without a GU‐rich sequence (miR‐186‐5p Mut) (Figure 5A, top).

Given that TLR8 is located at endosomal vesicles, we next examined whether exosomal miR‐186‐5p can reach endosomal TLR8 in HK2 cells. In this experiment, HK2 cells were incubated with miR‐186‐5p‐Cy5 enriched T‐EVs for 2 h. TLR8 and endosomes were then labeled with anti‐TLR8 antibody and Lyso‐Tracker Green, respectively. Co‐localization of anti‐TLR8 antibody and Lyso‐Tracker Green confirmed the endosomal localization of TLR8 in HK2 cells (Figure 5B). The fluorescent image of miR‐186‐5p‐Cy5 also showed that miR‐186‐5p‐Cy5 was internalized by HK2 cells and largely co‐localized with endosomal TLR8 (Figure 5B). As a main transcription factor of TLR8 signals downstream, NF‐*κ*B regulates the expression of various inflammatory cytokines, including TNF‐*α* and IL‐6, two cytokines that have been reported to induce injury of PTCs.^[^ ^27^ ^]^ We assessed whether miR‐186‐5p can affect NF‐*κ*B activity in HK2 cells by activating the TLR8 signaling pathway. In this experiment, we transfected an NF‐*κ*B reporter system into HEK293T cells and then incubated cells with *N*‐[1‐(2,3‐dioleoyloxy)propyl]‐*N*,*N*,*N* trimethylammonium methyl‐sulfate (DOTAP) or DOTAP formulation containing miR‐186‐5p or miR‐186‐5p Mut. NF‐*κ*B activity was detected with luciferase as previously described.^[^ ^25^ ^]^ We found that treatment with miR‐186‐5p but not miR‐186‐5p‐Mut strongly increased NF‐*κ*B activity (Figure 5C), as well as TNF‐*α* and IL‐6 expression (Figure 5D). Enhanced expression of TNF‐*α* and IL‐6 by miR‐186‐5p but not miR‐186‐5p‐Mut was completely abolished by TLR8 inhibitor enpatoran (Figure 5D), suggesting that the effect of miR‐186‐5p on eliciting inflammation is through activating TLR8 signal. Further analysis indicated that miR‐186‐5p but not miR‐186‐5p‐Mut increased TNF‐*α* and IL‐6 expression in a time (Figure 5E) and concentration‐dependent manner (Figure 5F), supporting that miR‐186‐5p does serve as a ligand for endosomal TLR8 and activate inflammatory cytokine expression. A previous study indicated that TLR7 and TLR8 differentially activated the IRF and NF‐*κ*B pathways.^[^ ^28^ ^]^ To clarify whether the NF‐*κ*B pathway is involved in TLR7/8‐mediated inflammatory activation, we examined the effect of NF‐*κ*B inhibitor Triptolide on miR‐186‐5p‐induced inflammatory cytokine expression. As shown in Figure 5G, Triptolide markedly blocked the inflammatory cytokine expression induced by miR‐186‐5p. In line with this, incubation with miR‐186‐5p but not miR‐186‐5p‐Mut resulted in time‐dependent HK2 cell apoptosis (Figure 5H).

**Figure 6:**
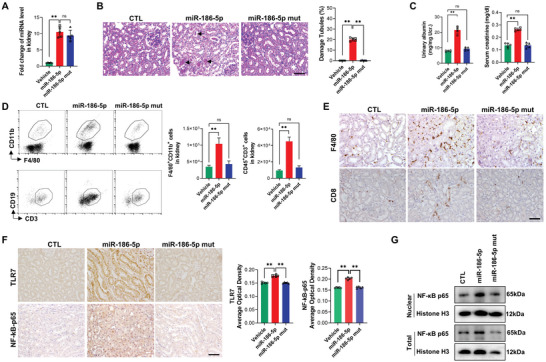
Injection of miR‐186‐5p initiates mouse renal inflammation via specific activating renal tubular TLR7 signal. A) kidney miR‐186‐5p level before and after miR‐186‐5p or miR‐186‐5p‐Mut injection. B) Mouse renal tubule damage quantitated by PAS. C) Proteinuria and creatinine level in mice on day 7 post‐injection of miR‐186‐5p or miR‐186‐5p‐Mut. D) Flow cytometry analysis of infiltration of macrophages (F4/80^+^CD11b^+^) and T cells (CD45^+^CD3^+^) in mice after miR‐186‐5p or miR‐186‐5p‐Mut injection. E) Tissue staining of macrophage and CD8 T cell infiltration in mouse kidney with miR‐186‐5p or miR‐186‐5p‐Mut treatment. F) Immunohistochemical detection (left) and analysis (right) of protein level of TLR7 and NF‐*κ*B‐p65 in mouse kidney tissues after miR‐186‐5p or miR‐186‐5p‐Mut treatment. G) Western blot analysis of nuclear and total NF‐*κ*B‐p65 level in HK2 cells transfected with miR‐186‐5p or miR‐186‐5p‐Mut. Scale bars, 50 µm. A,C,D) There were five mice per group and three tests for each mouse; B,E,F) 6–8 fields were analyzed for each mouse. A—D,F) Data were analyzed by unpaired two‐sided Student's *t*‐test. *, *p* < 0.05 and **, *p* < 0.01. ns, no significance.

To further validate the role of miR‐186‐5p in eliciting mouse renal inflammation by activating TLR7 signaling in renal tubular cells, we next directly administrated mice with synthetic miR‐186‐5p, miR‐186‐5p‐Mut or saline (vehicle) via tail vein. As shown in **Figure** **6**A, direct tail vein injection of miR‐186‐5p but not miR‐186‐5p‐Mut strongly increased the level of miR‐186‐5p in mouse renal tissues. Histopathological analysis revealed a significantly higher score in damage of tubules, particularly the brush border abscission of PTCs in mice administered with miR‐186‐5p compared to mice treated with vehicle or miR‐186‐5p‐Mut (Figure 6B). Treatment with miR‐186‐5p but not miR‐186‐5p‐Mut also increased creatinine levels (Figure 6C). Both flow cytometry analysis (Figure 6D) and immunohistochemical staining (Figure 6E) of immune cell infiltration indicated strong infiltration of F4/80^+^CD11b^+^ macrophages and CD8 T cells into kidney tissues of mice treated with miR‐186‐5p but not miR‐186‐5p‐Mut or saline. In line with this, the levels of TLR7 and NF‐*κ*B‐p65 expression in mouse renal tubules were markedly increased after direct injection of miR‐186‐5p but not miR‐186‐5p‐Mut (Figure 6F). Western blot analysis of cell fraction NF‐*κ*B‐p65 level in HK2 cells treated with miR‐186‐5p or miR‐186‐5p‐Mut further showed that treatment with miR‐186‐5p but not miR‐186‐5p‐Mut increased nuclear NF‐*κ*B‐p65 level (Figure 6G).

It has been widely shown that monocytes, as major innate immune cells, are activated under renal inflammation.^[^ ^29^ ^]^ We, therefore, examined the level of monocytes in mouse peripheral blood with or without injection of miR‐186‐5p or miR‐186‐5p Mut. As shown, blood monocyte level in mice injected with miR‐186‐5p but not miR‐186‐5p Mut was markedly increased (Figure S9A, Supporting Information, left). The elevation of blood monocyte level was also observed in ADR‐treated mice (Figure S9A, Supporting Information, right), as well as in FSGS patients (Figure S9B, Supporting Information).

### 2.4 Tlr7‐Deficient Mice Resist to Renal Inflammation and Tissue Injury Induced by miR‐186‐5p or ADR Treatment

**Figure 7:**
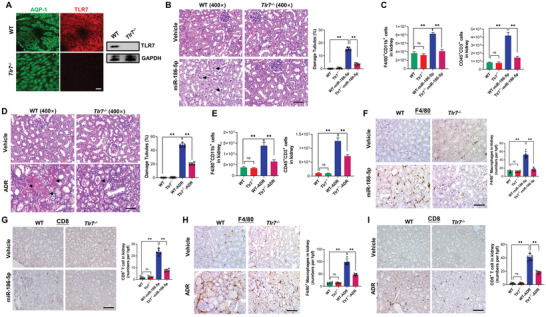
TLR7‐deficient mice resist renal inflammation and tissue injury induced by miR‐186‐5p or ADR administration. A) Immunofluorescence labeling (left) and western blot analysis (right) of TLR7 in mouse kidney tissues. B,D) Renal tubular injury induced by B) miR‐186‐5p or D) ADR treatment in WT and Tlr7^−/−^ mice. C,E) Flow cytometry analysis of renal infiltration of macrophages (F4/80^+^CD11b^+^) and T cells (CD45^+^CD3^+^) induced by C) miR‐186‐5p or E) ADR treatment in WT and Tlr7^−/−^ mice. F,G) Tissue staining of F) macrophage and G) CD8 T cell infiltration in WT and Tlr7^−/−^ mouse kidney with or without miR‐186‐5p treatment. H,I) Tissue staining of H) macrophage and I) CD8 T cell infiltration in WT and Tlr7^−/−^ kidney with or without ADR treatment. Scale bars, 50 µm. B–I) There were five mice per group; B,D,F—I) 6–8 fields were analyzed for each mouse. B—I) Data were analyzed by unpaired two‐sided Student's *t*‐test. **, *p* < 0.01. ns, no significance.

To validate the role of TLR7 signaling in miR‐186‐5p‐induced kidney injury, we examined the effect of miR‐186‐5p on renal inflammation and tissue injury in Tlr7^−/−^ mice.^[^ ^25^, ^30^ ^]^ In this experiment, WT and Tlr7^−/−^ mice were treated with or without miR‐186‐5p or ADR, respectively. Both immunofluorescence labeling and western blot analysis confirmed Tlr7 deficiency in mouse renal tubules (**Figure** **7**A). As shown, WT mice treated with synthetic miR‐186‐5p (WT‐miR‐186‐5p) (Figure 7B) or ADR (Figure 7D) displayed significant renal tubular cell damage, whereas either miR‐186‐5p or ADR treatment failed to induce renal tubular cell damage in Tlr7^−/−^ mice. Compared to WT mice, Tlr7^−/−^ mice treated with miR‐186‐5p (Tlr7^−/−^‐miR‐186‐5p) (Figure 7C) or ADR (Tlr7^−/−^‐ADR) (Figure 7E) displayed markedly less renal infiltration of F4/80^+^CD11b^+^ macrophages and CD45^+^CD3^+^ T cells assessed by flow cytometry. In agreement with this, renal infiltration of F4/80^+^ macrophages (Figure 7F,H) and CD8 T cells (Figure 7G,I) induced by miR‐186‐5p (Figure 7F,G) or ADR (Figure 7H,I) treatment was largely abolished in Tlr7^−/−^ mice. These results collectively suggest that the TLR7 signaling pathway is involved in mouse renal inflammatory responses and tissue injury induced by exosomal miR‐186‐5p or ADR.

To specifically deplete TLR7 signaling in mouse kidneys especially renal tubular cells, we directly injected AAV‐TLR7 shRNA (5ʹ‐GCCCTTTACCTGGATGGAAAC‐3ʹ) or scramble shRNA (Ctrl) into the mouse renal cortex as described previously.^[^ ^11^ ^]^ Briefly, 2 weeks prior to ADR treatment, mice were anesthetized by intraperitoneal injections of sodium pentobarbital (30 mg kg^−1^). After the mice were fully anesthetized, the renal was exposed via a flank incision. Then the adenovirus vectors were slowly injected into the renal cortex at six different sites (10 µL solution for each site) with a 31‐gauge needle. As shown in Figure S10A, Supporting Information, direct injection of AAV‐TLR7 shRNA strongly reduced renal tubular TLR7 expression. Reduction of renal tubular TLR7 by AAV‐TLR7 shRNA markedly attenuated renal injury induced by miR‐186‐5p (Figure S10B, Supporting Information). AAV‐TLR7 shRNA administration also inhibited kidney infiltration of F4/80^+^CD11b^+^ macrophages and CD8 T or CD45^+^CD3^+^ T cells assayed by flow cytometry (Figure S10C, Supporting Information) and immunohistochemical staining (Figure S10D,E, Supporting Information) induced by miR‐186‐5p.

### 2.5 Potential Role of TLR8 Signal Axis in Renal Inflammation in FSGS Patients

We next explored the potential role of the TLR8 signal axis in renal inflammation and tissue injury in FSGS patients. First, we compared the TLR8 expression level and infiltration of macrophages and CD8 T cells in renal tissues of FSGS patients and control kidney tissues. As shown in Figure S11A, Supporting Information, FSGS patients displayed a markedly enhanced TLR8 expression and NF‐*κ*B‐p65 in renal tubules compared to control, suggesting that TLR8‐mediated inflammatory signal may play a critical role in the development and progression of FSGS. This finding is in agreement with the previous report of restricted expression of TLR8 in the tubulointerstitial of nephropathy.^[^ ^26^ ^]^ Second, analysis of the NephroSeq online database (GSE108112) further showed that the renal tubular TLR8 level was significantly elevated in FSGS patients (Figure S11B, Supporting Information)^[^ ^31^ ^]^ and negatively correlated with patients’ GFR value (Figure S11C, Supporting Information).

## 3 Discussion

**Figure 8:**
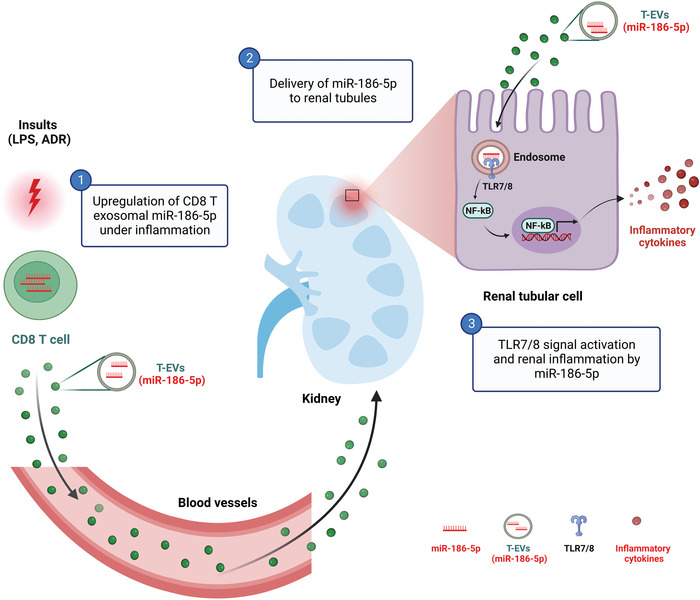
Schematic depiction of CD8 T cell exosomal miR‐186‐5p as a circulating pathogenic factor in causing renal inflammation and tissue injury via activating tubular TLR7/8 signal.

Through analyzing renal tissues of FSGS patients and WT or Tlr7‐deficient mice treated with ADR or miR‐186‐5p, we demonstrate that exosomal miR‐186‐5p secreted by activated CD8 T cells can initiate renal inflammation and tissue damage. As depicted in **Figure** **8**, our findings reveal for the first time the causative role of exosomal miR‐186‐5p as a circulating pathogenic factor in T cell‐mediated renal dysfunction.

Although different factors, including inflammation cytokines and reactive oxygen species, have been suggested to contribute to renal inflammation and tissue damage, the circulating factor that is responsible for T cell‐mediated FSGS remains undefined. Our results suggest that exosomal miR‐186‐5p derived from activated CD8 T cells is such a circulating factor. First, CD8 T cells expressed the highest level of miR‐186‐5p among all circulating cells, and miR‐186‐5p level in either CD8 T cells or CD8 T‐EVs was markedly increased under inflammatory stimulation, suggesting that CD8 T cells are a major source of exosomal miR‐186‐5p. Second, we detected the upregulation of renal tubular miR‐186‐5p but not pre‐miR‐186‐5p, implying that renal miR‐186‐5p was not due to de novo synthesis but from an extracellular source. This notion is validated by direct tracing exosome or exosomal miR‐186‐5p in mouse kidneys. Furthermore, intravenous injection of miR‐186‐5p but not miR‐186‐5p‐Mut initiating mouse renal inflammation and tissue injury confirms miR‐186‐5p as a circulating pathogenic factor to induce kidney dysfunction.

The miRNAs play an important role in modulating the function of various renal cells.^[^ ^32^ ^]^ In general, the function of a miRNA is executed via base‐pairing its mRNA target.^[^ ^33^, ^34^ ^]^ However, we found that T cell‐secreted miR‐186‐5p performed its function in a non‐classical mechanism in renal tubular cells and served as an endogenous ligand for renal tubular TLR7/8. This finding is in agreement with the notion that mature miRNA has a low affinity for AGO2, a crucial component required for the molecular biological function of miRNA.^[^ ^25^, ^35^ ^]^ Our results showed that miR‐186‐5p can directly bind to TLR7/8 through GU‐rich sequence and injection of synthetic miR‐186‐5p can initiate renal inflammation and tissue injury.

Various TLRs are involved in the development of severe autoimmune disease and inflammation in multiple organs, including the kidney.^[^ ^36^, ^37^ ^]^ TLRs can be activated by endogenous ligands released by self‐tissue or damaged cells in various disease conditions.^[^ ^38^ ^]^ For instance, HMGB1 can activate TLR4 and induce podocyte and tubular injury.^[^ ^39^ ^]^ However, the role of TLR7 (murine)/8 (human) in renal innate immunity and disease development is unclear. Through immunostaining and Western blot analysis, we showed that TLR8 was mainly localization at renal PTCs, with almost no or little expressed in podocytes, mesangial, and endothelial cells. This observation is consistent with the previous report by Conti et al. showing a highly restricted expression of TLR8 in the tubulointerstitial of LN patients.^[^ ^26^ ^]^ We further found that TLR8 was markedly upregulated in renal tissues of FSGS patients compared to that in para‐carcinoma tissues. Through analyzing the key elements of TLR8 signaling downstream, we confirmed that NF‐*κ*B and TNF‐*α* levels were increased in PTCs following TLR8 activation. In line with previous findings that TNF‐*α* induced PTCs apoptosis,^[^ ^40^ ^]^ our results suggest that TNF‐*α* may serve as an important inflammatory cytokine downstream of TLR8 in causing PTCs injury. As an RNA sensor, TLR7/8 preferentially binds to G(A)U‐rich of single strand RNA (ssRNA),^[^ ^41^, ^42^, ^43^ ^]^ including miRNA.^[^ ^25^, ^44^, ^45^, ^46^ ^]^ The activation of human TLR8 by such ssRNA led to a different spectrum of inflammatory diseases.^[^ ^36^, ^37^ ^]^ More importantly, as extracellular miRNAs can be transferred across organs, they can serve as a circulating factor to activate the TLR7/8 signal in different tissues. Two other miRNAs with GU‐rich motif, miR‐122, and let‐7b, were previously reported to trigger TLR7/8‐mediated lung inflammatory responses ^[^ ^25^ ^]^ and neurodegeneration,^[^ ^47^ ^]^ respectively. Our findings demonstrate that, via activating renal tubular TLR7/8 signaling, CD8 T cell‐derived exosomal miR‐186‐5p serves as a circulating factor in initiating T cell‐mediated renal inflammation and tissue injury. Supporting the role of TLR7 in T cell‐mediated renal dysfunction, Tlr7‐deficient mice displayed significantly less renal inflammation and tissue damage induced by ADR or miR‐186‐5p treatment.

Although FSGS has long been regarded as a disease with glomerular dysfunction, immerging evidence suggests that renal tubular cell injury also contributes to disease development and progression.^[^ ^48^ ^]^ Renal tubular injury may even occur earlier than glomerular dysfunction,^[^ ^49^ ^]^ and cell injury molecules produced by damaged renal tubules may result in damage to glomeruli.^[^ ^50^, ^51^ ^]^ Tan et al. reported that mitigating renal tubular injury via reducing MMP‐7 expression provided significant protection against glomerular injury and proteinuria.^[^ ^52^ ^]^ Through illustrating the effect of T cell exosomal miR‐186‐5p on activating renal tubular TLR7/8 signal axis and initiating renal inflammation and tissue damage, our results support the notion that renal tubular injury induced by T cell miR‐186‐5p can initiate or aggravate glomerular damage, and provide the miR‐186‐5p‐TLR7/8 signal axis as a novel mechanism underlying T cell‐mediated renal dysfunction. In conclusion, our study demonstrates that circulating miR‐186‐5p carried by CD8 T cell exosomes is a key causative factor of renal inflammation and tissue injury. Measurement of the exosomal miR‐186‐5p level in peripheral blood may serve as a novel tool to monitor the development and progression of T cell‐mediated renal dysfunctions such as FSGS. As depletion of circulating miR‐186‐5p or suppression of TLR7/8 signaling strongly reduces renal inflammation and tissue injury, targeting the miR‐186‐5p‐TLR7/8 signal axis provides an effective approach to mitigate T cell‐mediated renal dysfunction.

## 4 Experimental Section

### Study Design

The purpose of this study was to explore the mechanism underlying T cell‐mediated renal dysfunction, particularly FSGS. Continuing the previous cohort study identifying the correlation of high plasma levels of miR‐186‐5p with proteinuria in FSGS patients, the levels of miR‐186‐5p and pre‐miR‐186‐5p were first examined in various circulating immune and kidney cells under various inflammatory conditions including ADR‐induced kidney injury. This study found that the upregulation of miR‐186‐5p in injured kidney tissues was largely derived from CD8 T cell exosomes. Next, a cell tracing assay was performed and it was observed that T cell exosomal miR‐186‐5p was preferentially delivered into renal tubular cells but not the podocytes. To demonstrate the causative effect of T cell exosomal miR‐186‐5p on renal inflammation and tissue injury, synthetic miR‐186‐5p or miR‐186‐5p‐enriched exosomes derived from inflamed T cells were directly injected into mice via tail vein and the potential renal inflammation and tissue damage were monitored. The co‐localization of miR‐186‐5p and endosomal TLR7, as well as their interaction and downstream signal, were examined at cellular and tissue levels using miR‐186‐5p mutant or Tlr7‐knockout mice as the respective controls. To identify possible intervening approaches to mitigate renal inflammation and tissue injury induced by inflamed T cells or ADR treatment, a neutralization strategy was employed to deplete miR‐186‐5p in circulation and renal tubules and then monitored the degree of kidney dysfunction. Sample sizes were determined on the basis of pilot studies in humans and mice. The number of biological replicates for each experiment was indicated in the Figure legends and data acquisition was performed under single‐blind conditions.

### Human Samples, Patient Characteristics, and Clinical Features

Five FSGS patients who underwent renal biopsies at the National Clinical Research Center of Kidney Disease, Jinling Hospital (Nanjing University) were enrolled. The inclusion criteria were biopsy‐proven FSGS, proteinuria>3.5 g 24 h^−1^, serum creatinine ≤ 3 mg dL^−1^, and no corticosteroid or other immunosuppressors given at the time of enrollment. The exclusion criteria were secondary FSGS, family history of kidney disease, hepatitis virus or human immunodeficiency virus infection, malignant tumors, liver, heart, or hematopoietic system disease. Blood samples from five healthy volunteers, who had no known history of kidney diseases, were obtained as controls. The control kidney tissues from nephrectomy patients (*n* = 5) with renal carcinoma were obtained from this center's renal biorepository. Para‐cancerous kidney tissues from nephrectomy patients exhibited no clinical features of renal dysfunction. Table S1, Supporting Information lists the demographics and clinical characteristics of these patients and control. Other 64 FSGS patients were enrolled for analyzing the correlation between the number of leukocytes with plasma miR‐186‐5p level or kidney injury. To test the specific elevation of miR‐186‐5p in active CD8 T cells, the blood drawn from five healthy donors (each 2 mL) was divided into two parts and treated with 200 ng mL^−1^ LPS or saline (control) at 37 °C for 6 h. After the removal of red blood cells, white blood cells (CD19 B cells, CD4 T cells, CD8 T cells, CD14 monocytes, CD16 neutrophils, and CD56 NKs) were sorted out using a flow cytometer (Moflox XDP, Beckman Coulter, USA) and miR‐186‐5p expression in different cell population was assessed by qRT‐PCR. All study protocols involved human sample collection and were approved by the Institutional Review Board at Jinling Hospital Affiliated with Nanjing University School of Medicine. Written informed consent was obtained from each individual before enrollment.

### Mouse Model

All animal procedures were approved by the Animal Care Committee of Nanjing University (Nanjing, China) (IACUC‐2212002). Male BALB/c or C57BL/6 mice were purchased from the Model Animal Research Center, Nanjing University (Nanjing, China) and bred in specific pathogen‐free conditions. To construct an ADR‐induced kidney injury mouse model, 8‐week‐old micewere administrated intravenously with ADR (10 mg kg^−1^ for BALB/c or 20 mg kg^−1^ for C57BL/6 mice^[^ ^20^, ^53^ ^]^) or sterile saline administration serving as vehicle control. Mice were euthanized 4 weeks following ADR treatment and the urine, blood, and renal tissue were collected. To establish the mouse T lymphocyte activation model, 8‐week‐old C57BL/6 mice were administrated intraperitoneally with 10 mg kg^−1^ LPS (Sigma‐Aldrich, L2762) with sterile saline administration serving as vehicle control. To verify the miR‐186‐5p‐induced renal injury, 10 nmol of synthetic miR‐186‐5p (Genepharma, Shanghai, China), miR‐186‐5p‐Mut, or vehicle were dissolved in 150 µl of PBS and then injected into C57BL/6 mice via tail vein every other day for 1 week. To decrease the level of miR‐186‐5p in the LPS mouse model, 10 nmol of synthetic cholesterol‐modified miR‐186‐5p ASO (AGCCCAAAAGGAGAATTCTTTG) or negative control (both from RiboBio) were dissolved in 150 µL of PBS with or without LPS and then injected into C57BL/6 mice via tail vein. Mice were euthanized 3 days after treatment with LPS alone or LPS plus miR‐186‐5p ASO. Tlr7^−/−^ mice were acquired from the Jackson Laboratory (Bar Harbor, Maine, USA) and were backcrossed with C57BL/6 mice over 10 generations.

### Urine Albumin and Creatinine Detection

The mouse urine samples were collected in metabolic cages and centrifuged at 3000× g for 10 min to discard the precipitation in urine. The albumin and creatinine levels were separately measured by using Albuwell M (Exocell, 1011) and the Creatinine Companion kit (Exocell, 1012) according to the manufacturer's protocol.

### Cell Culture, Treatment, and Apoptosis Assay

HK2 cells were purchased from American Type Culture Collection, and cultured in F12/DMEM supplemented (Gibco, A4192001) with 10% fetal bovine serum (Gibco, 10099141). CD8 T cells were obtained in the blood of FSGS patients and healthy control by flow cytometer (Moflox XDP, Beckman, USA), and proliferation and activation by 5 µg mL^−1^ anti‐CD3/CD28 antibody plus 100 U mL^−1^ rIL‐2 (Gibco, PHC0027) in the presence of 10% autologous serum (EVs‐free). Cells were incubated at 5% CO_2_ and 37 °C in a water‐saturated atmosphere. To analyze the inflammatory cytokines secretion, HK2 cells were seeded into 6‐well culture plates for 24 h. At 70% confluence, HK2 cells were treated for different times (0, 6, 12, and 24 h) or at different concentrations (0, 2, 4, 8, and 10 µg mL^−1^) of miR‐186‐5p, miR‐186‐5p Mut, or vehicle. To suppress the expression of inflammatory cytokines, HK2 cells were treated with NF‐*κ*B inhibitor triptolide (30 nM; MedChemExpress, HY‐32735) or TLR8 inhibitor enpatoran (100 nM; MedChemExpress, HY‐134581) alone or plus 10 µg mL^−1^ miR‐186‐5p. After 24 h incubation, the culture medium was centrifuged (1000× g, 10 min, 4 °C) to obtain the supernatant. HK2 cell apoptosis was assessed by surface labeling with fluorescent annexin V and PI kits (Vazyme, A211‐01) according to the manufacturer's instructions. Briefly, 2 × 10^5^ cells were dissociated by trypsin solutions (Gibco, 15050065), washed with ice‐cold PBS twice, and collected by centrifugation at 1800 rpm for 5 min. Cells were then resuspended in 100 µl binding buffer and incubated with 5 µl FITC‐annexin V and PI staining solution for 15 min in the dark. The percentage of apoptosis cells was evaluated by flow cytometry and analyzed by FlowJo_V10 software (BD Bioscience, USA).

### Immunohistochemistry and Periodic Acid‐Schiff Staining

For periodic acid‐Schiff staining, kidney tissues from mice or human patients were fixed in 4% paraformaldehyde buffer for several days, embedded in paraffin wax, and processed for sectioning (2 µm). For immunohistochemistry analysis, after mice were anesthetized, the kidneys were isolated and immediately fixed in 4% paraformaldehyde. Tissues were embedded in paraffin wax and performed with immunohistochemistry analysis as previously described.^[^ ^54^ ^]^ In brief, tissue sections were incubated with 1% BSA for 30 min at room temperature, and then incubated with primary antibodies overnight. After three washes with PBS, tissue sections were labeled with secondary peroxidase‐conjugated antibodies for 40 min. The signal was generated using the DAB kit (Vector Laboratories, California, USA, SK‐4100). The images were captured by Nikon Eclipse E800 microscopy (Nikon, Japan). In this experiments, primary antibodies against F4/80 (1:200, Cell Signaling Technology, 70076), mouse CD8 (1:200, Cell Signaling, 98941S), TLR8 (1:100, Beyotime Biotechnology, AF8190), TLR7 (1:100, Beyotime Biotechnology, AF0300), and NF‐*κ*B‐p65 (1:400, abcam, ab16502) were used. All histologic studies were conducted in a blinded manner by experienced renal pathologists as previously described.^[^ ^55^, ^56^ ^]^ The images were examined semi‐quantitatively by Image‐Pro Plus 6.0 software (Meyer Instruments, Houston, USA). Six high‐magnitude fields from each slide were analyzed.

### Electron Microscopy and Quantification of Foot Process Effacement

Fresh renal cortex tissues (1 mm^3^ pieces) pre‐fixation in 2.5% glutaraldehyde for 4 h and post‐fixed in phosphate‐buffered 2% osmium tetroxide for 2 h. After dehydration in acetone and ethanol, the samples were further embedded in epoxy resin. Ultrathin sections (80–90 nm) were collected on copper grids, and stained with 5% uranyl acetate for 15 min followed by 0.1% lead citrate for 5 min. The sections were viewed on a Hitachi 7500 transmission electron microscope (Hitachi Co., Japan). The podocyte foot process width was quantified using Image J as previously reported.^[^ ^57^ ^]^

### Immunofluorescence

Kidneys were frozen in OCT compound in liquid nitrogen and sectioned at 5 µm thickness using Leica CM1950. The sections were blocked with bovine serum albumin, and then incubated with primary antibodies overnight at 4 °C. Primary antibodies against TLR7 (1:100, Beyotime Biotechnology, AF0300), PODXL (1:1000, Invitrogen, 39–3800), AQP‐1 (1:1000, Abcam, ab168387), or F4/80 (1:200, Cell Signaling Technology, 70076) were used. Alexa Fluor 488 goat anti‐rabbit IgG (H+L) (1:1000, Invitrogen, A32731), Alexa Fluor 488 goat anti‐mouse IgG (H+L) (1:1000, Invitrogen, A28175), and Alexa Fluor 633 goat anti‐rabbit IgG (H+L) (1:1000, Invitrogen, A‐21070) secondary antibodies were used. Then the sections were stained with DAPI (Invitrogen, D1306) and mounted in Prolong Diamond Antifade Mountant (Life Technologies, P36961). For co‐localization analysis of TLR8 and miR‐186‐5p, HK2 cells were seeded into 6‐well culture plates for 24 h. After 70% confluence, HK2 cells were treated with 10 µg mL^−1^ miR‐186‐5p‐Cy5 enriched T‐EVs for 2 h washed three times with Hank's dilution, and incubated with LysoTracker Green (Beyotime Biotechnology, C1047S, 1:25 000 dilution) for 30 min. After three washes with Hank's solution, HK2 cells were incubated with TLR8 primary antibody (1:100, Beyotime Biotechnology, AF8190) for 2 h and consequently with Alexa Fluor 350 goat anti‐rabbit IgG (H+L) antibody (1:300, Beyotime Biotechnology, A0408). Images were obtained under a confocal microscope (LSM710, ZEISS, Germany) and fluorescence intensities were quantified using ImageJ software.

### Flow Cytometry

To analyze the infiltrated immune cells in mouse kidneys, the kidney was taken from mice, carefully removed the fascial layer by surgical instruments, and cut average into four parts. One‐quarter of the mouse kidney was used to dissociate into a single cell suspension using a gentleMACS dissociator (Miltenyi Biotec, Germany) and Multi‐Tissue Dissociation Kits (Miltenyi Biotec, 130‐110‐203) according to the manufacturer's protocol. The single cell suspension was collected by centrifugation at 1000× g for 10 min, and then incubated respective antibodies and detected by flow cytometer. The antibodies were obtained from indicated sources: PerCP conjugated to anti‐mouse CD45 (BD Biosciences, 557235), PE‐Cy7 conjugated to anti‐mouse CD3e (BD Pharmingen, 552774), APC conjugated to anti‐mouse F4/80 (BioLegend, 157305), FITC conjugated to anti‐mouse/human CD11b (BioLegend, 101205), FITC conjugated to anti‐mouse CD19 (BioLegend, 152404). To analyze T cells or monocytes in mouse peripheral blood, 100 µL peripheral blood was lysed by 1 mL lysing reagent (Solarbio, R1010) for 5 min and washed in cold PBS (10 mM HEPES, 150 mM NaCl, 2.5 mM CaCl_2_) twice. To analyze T cells or monocytes in mouse spleen, mouse spleen tissues were cut into small pieces (5–10 mm^3^) and dissociated into a single cell suspension using the piston plunger of a sterile 20 ml syringe. The released splenocytes were filtered by a 70 µm cell strainer and washed with PBS twice. The isolated splenocytes were then treated with lysing reagent (Solarbio, R1010) for 5 min followed by centrifugation (1000× g, 10 min) to collect cells. Cells were resuspended in PBS and incubated with respective antibodies. The following antibodies were used: PerCP conjugated to anti‐mouse CD45 (BD Biosciences, 557235), PE‐conjugated to anti‐mouse CD3e (BD Pharmingen, 553063), APC conjugated to anti‐mouse CD4 (BD Pharmingen, 553051), PE/cyanine7 conjugated to anti‐mouse CD8a (Biolegend, 100722), FITC conjugated to anti‐mouse Ly‐6C (Biolegend, 128005). To sort out different white blood cells, 2 mL of human peripheral blood was treated with 20 mL lysing reagent (Solarbio, R1010) and washed twice with PBS. White blood cells were then incubated with different antibodies, and sorted out by flow cytometer. The following antibodies were used: APC‐A750 conjugated to CD45 (Beckman Coulter, A79392), FITC/PE/PC5 conjugated to CD4/CD8/CD3 antibody cocktail (Beckman Coulter, IM1650), APC conjugated to anti‐human CD14 (BioLegend, 325608), ECD conjugated to CD19 (Beckman Coulter, A07770), Alexa Fluor 488 anti‐human CD16 (BioLegend, 302022), and PC7 conjugated to CD56 (Beckman Coulter, A21692). Flow cytometry data obtained from the above experiments were analyzed with FlowJo‐V10 software (BD Bioscience, USA). To sort or analyze the circulating T‐EVs in FSGS patients and healthy donors, various reagents were used to label EVs: PKH67 (Sigma‐Aldrich, PKH67GL), APC conjugated to mouse anti‐human CD3 (BD Pharmingen, 561810) and annexin V (BioLegend, 640907). Following the labeling, a gate for exosomes was created according to the positioning of fluorescent FluoSphere beads (Invitrogen, F8888).^[^ ^58^ ^]^

### Western Blot

Western blot analysis was performed as previously described.^[^ ^11^ ^]^ In brief, extracellular vesicles, cells, or kidney tissues were harvested by RIPA lysis buffer and the cell debris was depleted after 12 000× g centrifuge at 4 °C. The protein concentration was assessed using the enhanced BCA protein assay kit (Beyotime Biotechnology, P0010) according to the manufacturer's instruction. The supernatant was boiled at 100 °C for 5 min before being loaded into 12% polyacrylamide gel. Proteins resolved by SDS‐PAGE were transferred onto the polyvinylidene fluoride (PVDF) membranes (0.45 µm, Millipore, IPVH00010). The membranes were blocked in Tris‐buffered saline plus tween‐20 (TBS‐T, Solarbio, T1082) containing 5% BSA for 1 h and incubated with primary antibodies at 4 °C for overnight followed by six washes and incubation with secondary antibodies goat anti‐mouse IgG‐HRP or goat anti‐rabbit IgG‐HRP. The signals were assessed by enhanced chemiluminescence (NCM Biotech, P10100). The primary antibodies including CD3 (1:3000, Santa Cruz, sc‐1179), CD63 (1:3000, Santa Cruz, sc‐5275), TLR7 (1:1000, Beyotime Biotechnology, AF0300), GAPDH (1:5000, Santa Cruz, sc‐32233), histone‐H3 (1:5000, Proteintech,17168‐1‐AP), NF‐*κ*B p65(1:2000, Abcam, ab16502), and HRP‐conjugated secondary antibodies (1:5000, Beyotime Biotechnology, A0208) were used. Uncropped scans of all gels are provided in the Supporting Information.

### RNA Isolating and Quantitative RT‐PCR

Total RNA from kidney tissue or cells was extracted by TRIzol Reagent (Invitrogen, 15596018) following the manufacturer's instructions. The 100 µL plasma total RNA was extracted by 900 µL TRIzol LS Reagent (Invitrogen, 10296010) following the manufacturer's instructions. To detect the level of miRNA, stem‐loop RT‐qPCR assays using miRNA‐specific primer were performed as described previously.^[^ ^25^ ^]^ In brief, total RNA was used to reverse transcribed into cDNA using the miRNA‐specific stem‐loop RT primers (RT primers: GTCGTATCCAGTGCAGGGTCCGAGGTATTCGCACTGGATACGACAGCCCA), reverse transcriptase, and reverse transcription buffer, according to the manufacturer's instructions (Vazyme, MR101‐01). Subsequently, real‐time PCR was executed with the cDNA using miR‐186‐5p‐specific primers (Forward primers: CGCGCAAAGAATTCTCCTTT; Reverse primers: AGTGCAGGGTCCGAGGTATT) and QuantStudio 3 Real‐Time PCR System (Agilent Technologies, USA). To detect the level of pre‐miR‐186‐5p, reverse transcription PCR (RT primers: GTCGTATCCAGTGCAGGGTCCGAGGTATTCGCACTGGATACGACAGCCCA) and real‐time PCR (Forward primers: CGCGCGCGCAAAGAATTCTCCTTT; Reverse primers: ATCCAGTGCAGGGTCCGAGG) were performed. To calculate the absolute expression levels of miR‐186‐5p, synthetic miR‐186‐5p oligonucleotides (Genepharma, Shanghai, China) at known concentrations were used to build a standard curve and formula, then the absolute amount of miR‐186‐5p was calculated.

### Extracellular Vesicle Isolation and Characterizer Identification

EVs were isolated from cell culture supernatant or plasma via ultracentrifugation by ultracentrifuge (Optima L‐100XP, Beckman Coulter, USA) as previously described.^[^ ^25^ ^]^ Briefly, the cell debris was discarded through sequential centrifugation at 300× g for 5 min, 1200× g for 10 min, and 10 000× g for 30 min at 4°C, then centrifuged at 100 000× g for 70 min at 4 °C to obtain EVs. Finally, EVs were dispersed in cold PBS for transient storage or stored at −80°C for long‐term storage. EV surface markers were detected by Western blot.

### Tracing Assay

To test whether T‐EVs or miR‐186‐5p could enter renal tissue, a tracing assay was performed with different labeling strategies. T‐EVs were then collected from the culture medium and labeled with 5 µM DiI (Beyotime Biotechnology, C1036) prior to injection into mice via the tail vein. Firstly, the DiI‐labeled CD8 T‐EVs were traced in mouse kidneys 3 h following the injection. Secondly, to test whether renal inflammation and tissue injury increase the homing of CD8 T‐EVs to the kidney, the distribution of intravenously injected DiI‐labeled CD8 T‐EVs in mouse kidneys with or without ADR treatment for 4 weeks was analyzed. To trace the cellular distribution of miR‐186‐5p, synthetic 10 nM miR‐186‐5p‐Cy5 was also directly injected into mice via the tail vein.

### Intravenous Injection of a Recombinant Adeno‐Associated Viral‐Based Mir‐186‐5p Sponge

To specifically decrease miR‐186‐5p level in mouse kidneys and plasma, a recombinant adeno‐associated viral (AAV)‐based miR‐186‐5p sponge (Hanbio) was constructed and injected into mice (1 × 10^12^ copies per mouse) via tail vein. Briefly, the chemically synthesized miR‐186‐5p sponge sequence was digested and cloned into the pHBAAV‐CMV‐MCS vector (Hanbio). The pHBAAV‐miR‐186‐5p sponge‐CMV‐Luc and pHelper, pAAV‐RC were co‐transfected into AAV‐293 cells using LipoFiter transfection reagent (Hanbio) to generate the recombinant AAV. At 72 h post‐transfection, cells were collected and subjected to repeated freezing and thawing with liquid nitrogen three times to collect the supernatant and then digested with Benonase enzyme. The supernatant was purified with a Biomiga brand purification column, and the virus was stored at −80 °C after sub‐packaging. All oligonucleotide sequences were confirmed by nucleotide sequencing. The titers of the recombinant adenovirus were determined by RT‐PCR using vector‐specific primers. Eight‐week‐old BALB/c male mice were injected with AAV vectors (1 × 10^12^ copies per mouse) via tail vein prior to ADR or vehicle treatment.

### AAV‐TLR7 shRNA–Expressing Adeno‐Associated Viral Preparation and Intrarenal Injection

TLR7‐specific shRNA (5ʹ‐GCCCTTTACCTGGATGGAAAC‐3ʹ) and scramble shRNA were subcloned into a pHBAAV‐U6‐MCS‐CMV‐Luc vector (Hanbio), respectively. The pHBAAV‐TLR7 shRNA‐CMV‐Luc and pHelper, and pAAV‐RC were co‐transfected into AAV‐293 cells to generate the recombinant AAV. The titers of the recombinant AAV were determined by RT‐PCR and vector‐specific primers. The virus was injected into the mouse renal cortex according to previously described methods.^[^ ^11^ ^]^ Briefly, 2 weeks prior to ADR treatment, mice were anesthetized by intraperitoneal injections of sodium pentobarbital (30 mg kg^−1^). After the mice were fully anesthetized, the renal was exposed via a flank incision. The adenovirus vectors were then slowly injected into the renal cortex at six different sites (10 µl solution for each site) with a 31‐gauge needle.

### RNA‐Binding Protein Immunoprecipitation Assay

HK2 cells were incubated with the synthetic miRNAs for 30 min. The cells were washed by the ice‐cold PBS twice and digested by trypsin (Gibco, 15050065), and then collected and lysed with 200 µL of lysis buffer (including 2 µL protease inhibitor cocktail, 0.1 µL 1 M DTT and 0.8 µL ribonuclease inhibitor) for 15 min in ice‐bath. Lysates were collected at 16 000× g at 4 °C for 10 min and immunoprecipitated with anti‐TLR8 antibody (1:100, Beyotime Biotechnology, AF8190) or normal IgG (1:100, Beyotime Biotechnology, A7001) followed by Protein A/G beads (Beyotime Biotechnology, P2179S). After washing the RNA from the beads, the binding RNA was extracted with TRIzol (Invitrogen, 15596026).

### NF‐*κ*B Activity Assay

NF‐*κ*B activity was assessed as previously described.^[^ ^25^ ^]^ In brief, 1 × 10^6^ HEK293T cells were cultured in 12‐well culture plates, and each well was subsequently transfected with 0.1 µg of an NF‐*κ*B luciferase reported plasmid, 0.1 µg of a *β*‐gal expression plasmid (negative control), and 0.5 µg of a TLR8 overexpression plasmid using Lipofectamine 2000 (Invitrogen, 11668019). After 24 h of cell culture, the HEK293T cells were treated with miRNA (5 µg ml^−1^) complexed with DOTAP for another 12 h and analyzed via luciferase (Promega).

### Enzyme‐Linked Immunosorbent Assay

The concentration of cytokines including TNF‐*α* and IL‐6 in the cell culture supernatant was assessed by the human ELISA Kit (both from Multi Sciences) according to the manufacturer's instructions.

### Cellular Fractionation Assay

The separation of nuclear and cytoplasmic fractions was performed using PARIS kits (Life Technologies, AM1921) according to the manufacturer's instructions. Briefly, after transfection with scramble RNA, miR‐186‐5p or miR‐186‐5p Mut, HK2 cells were collected and washed twice with ice‐cold PBS. Cells were then divided into two parts. One part was lysed with ice‐cold cell disruption buffer to obtain the total fraction and the other part was treated with equal volume cell fractionation buffer and kept on ice for 5 min. The mixture was centrifuged at 500× g for 5 min at 4 °C to obtain the cytoplasmic fraction, while the pellets were lysed with ice‐cold cell disruption buffer on ice for 30 min and then spun at 17 000× g for 10 min to pellet insoluble debris, yielding nuclear fraction. The enrichment cytoplasmic or nuclear fraction in isolated products were assessed by Western blot.

### In Vivo CD8 T Cell Depletion

To deplete mouse CD8 T cells, mice were injected with 100 µg per mouse anti‐CD8 mAb (rat IgG2b mAb, clone 2.43; BioXCell, BE0061) or isotype control mAb (rat IgG2b mAb, clone LTF‐2; BioXCell, BE0090) via tail vein 1 day prior to ADR treatment. After ADR administration, mice were injected with anti‐CD8 mAb on days 2, 7, 12, 17, and 22. Mice were euthanized 28 days following ADR treatment and the urine, blood, and renal tissue were collected for examination. Depletion of mouse blood and spleen CD8 T cells was confirmed by flow cytometry.

### Statistical Analysis

Data were obtained from at least three independent experiments and expressed as group means ± SD. Statistical differences between samples or groups were assessed by two‐way ANOVA with Dunnett's multiple comparisons test (Figure 5E,F), Spearman's correlation test (Figure S9 and Table S2, Supporting Information) or two‐tailed Student's *t‐*test (others). The sample number (*n*), independent biological experimental repeats, and statistical analysis details used are stated in the Figures or Figure legends. Statistical analysis was performed in GraphPad Prism 9.0. *p*‐value<0.05 was considered statistically significant.

### Ethics Approval and Consent to Participate

The collection of biospecimens was approved by the Institutional Review Board of Jinling Hospital (Nanjing, China) (2016NZGKJ‐041). All participants signed informed consent on using clinical specimens for medical research.

## Conflict of Interest

The authors declare no conflict of interest.

## Author Contributions

X.X. And S.Q. contributed equally to this work. K.Z. and Z.L. designed the study; X.X., S.Q., and M.Z. performed the experiments and data analysis; C.Z. enrolled the patients and clinical data acquisition; G.R. contributed to sample collection; S.Q., W.Q., and H.B. provided Tlr7^−/−^ mice and study materials; K.Z. and X.X. wrote the manuscript; K.Z., Z.L., and L.L. edited and reviewed the manuscript. All authors read and approved the final version of the manuscript.

## Supporting information

Supporting Information

## Data Availability

The data that support the findings of this study are available from the corresponding author upon reasonable request.
