# Supplementary material for: CD8 T Cell‐Derived Exosomal miR‐186‐5p Elicits Renal Inflammation via Activating Tubular TLR7/8 Signal Axis
Source: Adv Sci (Weinh). 2023 Jul 3;10(25):2301492. doi: 10.1002/advs.202301492 (PMC10477851; doi:10.1002/advs.202301492)

ADVANCED SCIENCE
Open Access

Supporting Information

for *Adv. Sci.*, DOI 10.1002/advs.202301492

CD8 T Cell-Derived Exosomal miR-186-5p Elicits Renal Inflammation via Activating Tubular TLR7/8 Signal Axis

Xiaodong Xu, Shuang Qu, Changming Zhang, Mingchao Zhang, Weisong Qin, Guisheng Ren, Hao Bao, Limin Li, Ke Zen and Zhihong Liu**

Supplementary data for

CD8 T cell-derived exosomal miR-186-5p elicits renal inflammation via activating tubular TLR7/8 signal axis

Xiaodong Xu[1†], Shuang Qu[2†], Changming Zhang[1], Mingchao Zhang[1], Weisong Qin[1], Guisheng Ren[1], Hao Bao[1], Limin Li[2], Ke Zen[3]*, Zhihong Liu[1]*

File list:

1. Supplementary Table 1
2. Supplementary Table 2
3. Supplementary Figure 1
4. Supplementary Figure 2
5. Supplementary Figure 3
6. Supplementary Figure 4
7. Supplementary Figure 5
8. Supplementary Figure 6
9. Supplementary Figure 7
10. Supplementary Figure 8
11. Supplementary Figure 9
12. Supplementary Figure 10
13. Supplementary Figure 11
14. Raw WB data

Supplementary Table 1. Characteristics of individuals enrolled in the study. Data were presented as group means±SD. M, male. F, female. n.d., not determined.

	**FSGS Patients (n = 5)**	**Healthy control (n = 5)**
Sex	3 M / 2 F	3 M / 2 F
Age, yr	32.2 ± 4.55	29.8 ± 1.64
Serum creatinine, mg/dL	1.34 ± 0.48	0.60 ± 0.16
Serum albumin, g/L	21.4 ± 3.02	50.16 ± 3.76
Proteinuria, g/24 h	9.01 ± 2.12	n.d.

Supplementary Table 2. The correlation of T cells number with kidney injury in FSGS patients. Patients (*n* = 64) were evaluated by Spearman's correlation test. Data were presented as group means±SD. R, Pearson correlation coefficient. *, $P < 0.05$.

**Correlation/**	**Total T cells number**		**CD4 T cells number**		**CD8 T cells number**	
	R	*P*	*R*	*P*	*R*	*P*
Proteinuria (g/24 h)	0.089	0.145	0.014	0.823	0.152	0.012*
Albumin (g/L)	0.029	0.631	0.08	0.186	-0.049	0.421
Creatinine (mg/dL)	-0.069	0.251	-0.076	0.206	-0.03	0.618
Acute tubulointerstitial lesions	0.08	0.18	-0.018	0.768	0.161	0.007*
Chronic tubulointerstitial lesions	0.084	0.16	0.052	0.381	0.135	0.023*

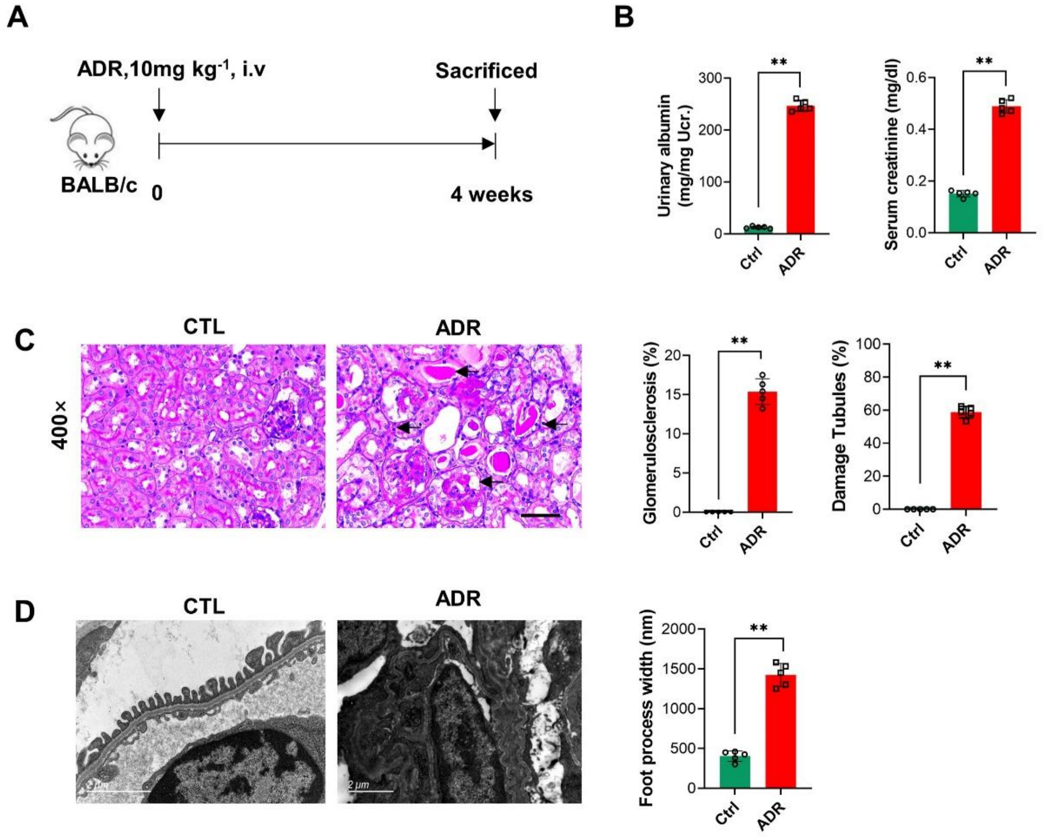

Supplementary Figure 1. ADR-induced mouse renal injury model. A) Schematic of ADR-induced renal injured mouse model. B) Proteinuria and serum creatinine levels in mice with or without ADR treatment. C) Histopathological staining of mouse renal tissues with or without ADR treatment. Scale bar, 50μm. D) TEM images of renal cortex tissues with or without ADR treatment. Scale bar, 2μm.There were 5 mice per group (B-D); 6-8 fields were analyzed for each mouse (C-D). Data were analyzed by unpaired two-sided Student's *t* test (B-D) and presented as group means ± SD. **, $P < 0.01$.

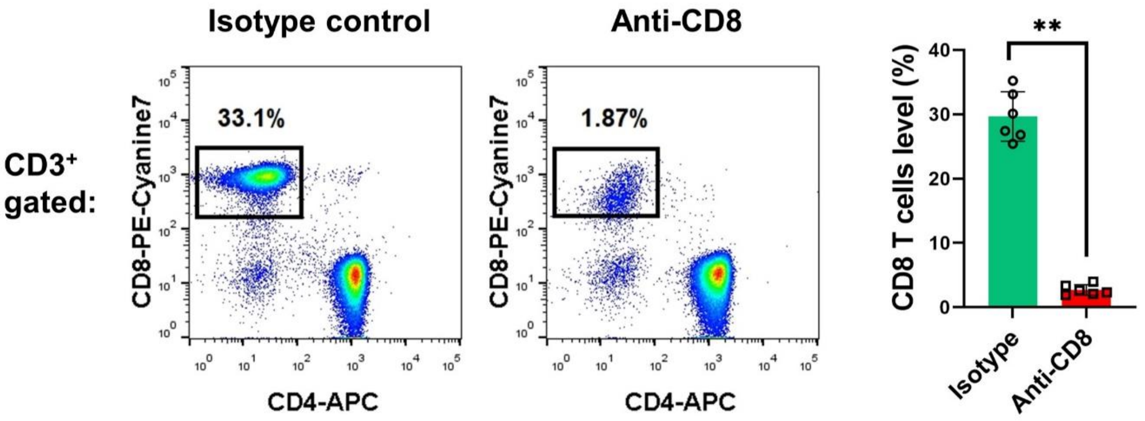

Supplementary Figure 2. **Depletion of mouse CD8 T cells by anti-CD8 mAb assayed by flow cytometry.** To deplete mouse CD8 T cells, mice were injected intraperitoneally with anti-CD8 mAb (rat IgG2b mAb, clone 2.43; BioXCell) or isotype control mAb (100μg each per mouse) 1 day prior to ADR treatment, and then injected with antibody on day 2, 7, 12, 17 and 22. Mice were euthanized 28 days after ADR treatment. CD8 T cell level in peripheral blood was assayed by flow cytometry. There were 6 mice per group. Data were analyzed by unpaired two-sided Student's *t* test and presented as group means ± SD. **, $P < 0.01$.

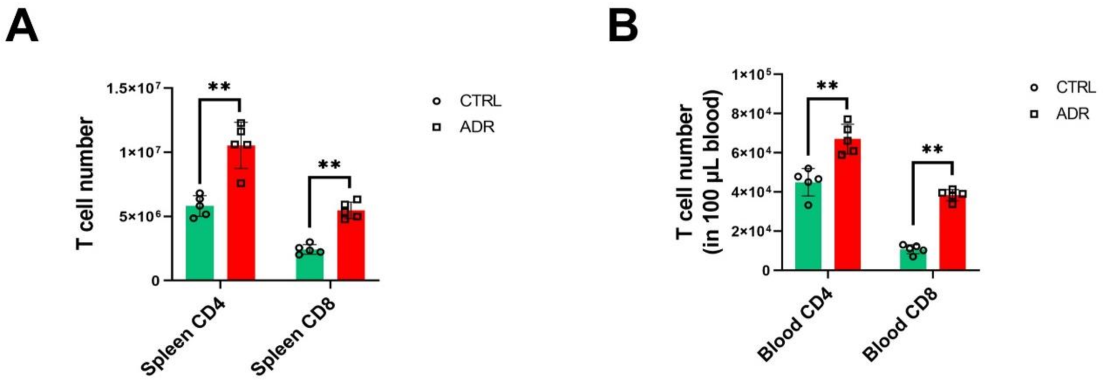

Supplementary Figure 3. Increase of CD8 and CD4 T cell numbers in ADR-treated mice. A) Spleen CD4 and CD8 T cell number in mice treated with or without ADR. B) CD4 and CD8 T cell number in peripheral blood of mice treated with or without ADR. There were 5 mice per group (A-B). Data were analyzed by unpaired two-sided Student's *t* test (A-B) and presented as group means ± SD. **, $P < 0.01$.

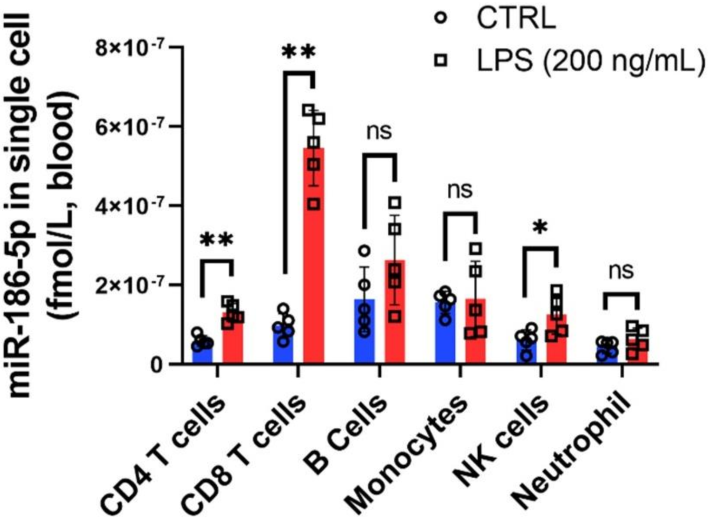

Supplementary Figure 4. Strong increase of miR-186-5p level in human CD8 T cells but not other white blood cells under LPS stimulation. To assess the specificity of cellular secretion of miR-186-5p, blood drawn from 5 healthy donors (2 mL each) was divided into two parts, and then treated with 200 ng/mL LPS or saline (control) at 37°C for 6 h. After removal of red blood cells, CD19 B cells, CD4 T cells, CD8 T cells, CD14 monocytes, CD16 neutrophil and CD56 NK cells were sorted out by flow cytometry and miR-186-5p level in different cell populations was assayed by qRT-PCR. Data from three independent experiments were analyzed by unpaired two-sided Student's *t* test and presented as group means ± SD. *, $P < 0.05$; **, $P < 0.01$; ns, no significant.

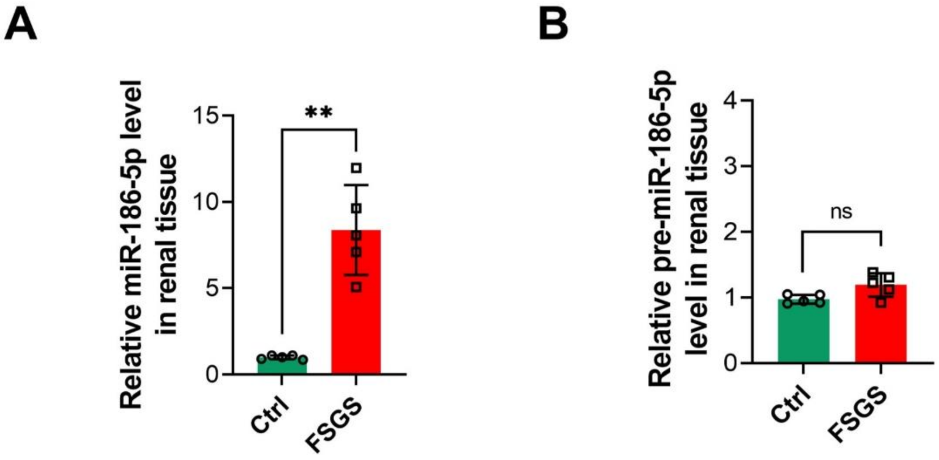

Supplementary Figure 5. The levels of miR-186 and pre-miR-186 in kidney tissues from 5 FSGS patients and control para-carcinoma (HC) kidney tissues. A-B) Renal miR-186 (A) and pre-miR-186 (B) level in FSGS patients and controls. There were 5 patients or healthy donors (Ctrl) in each group. Data were analyzed by unpaired two-sided Student's *t* test and presented as group means ± SD. **, $P < 0.01$. ns, no significant.

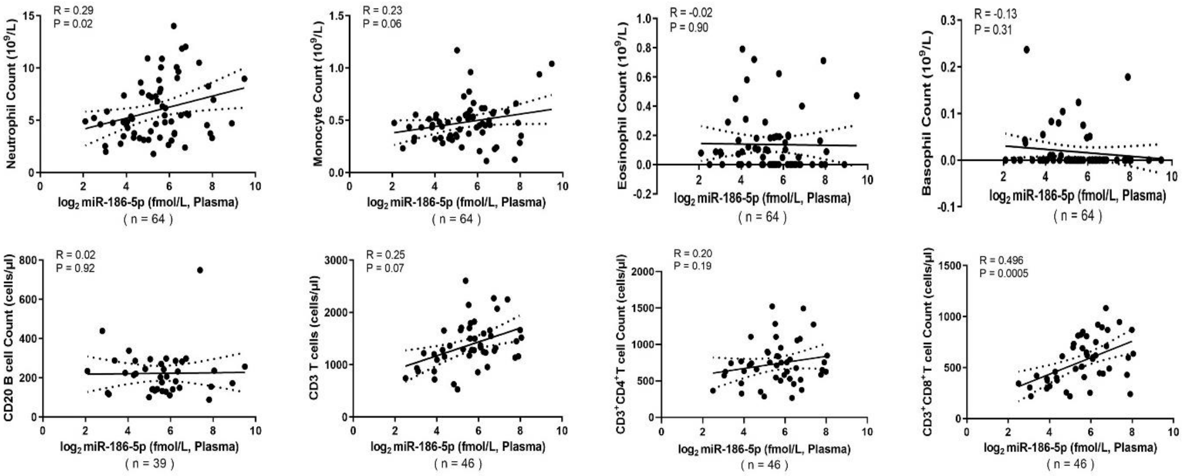

Supplementary Figure 6. The correlation between circulating miR-186-5p levels and various leukocyte counts in plasma of FSGS patients. R, Pearson correlation coefficient. *P* value was evaluated by Spearman's correlation test.

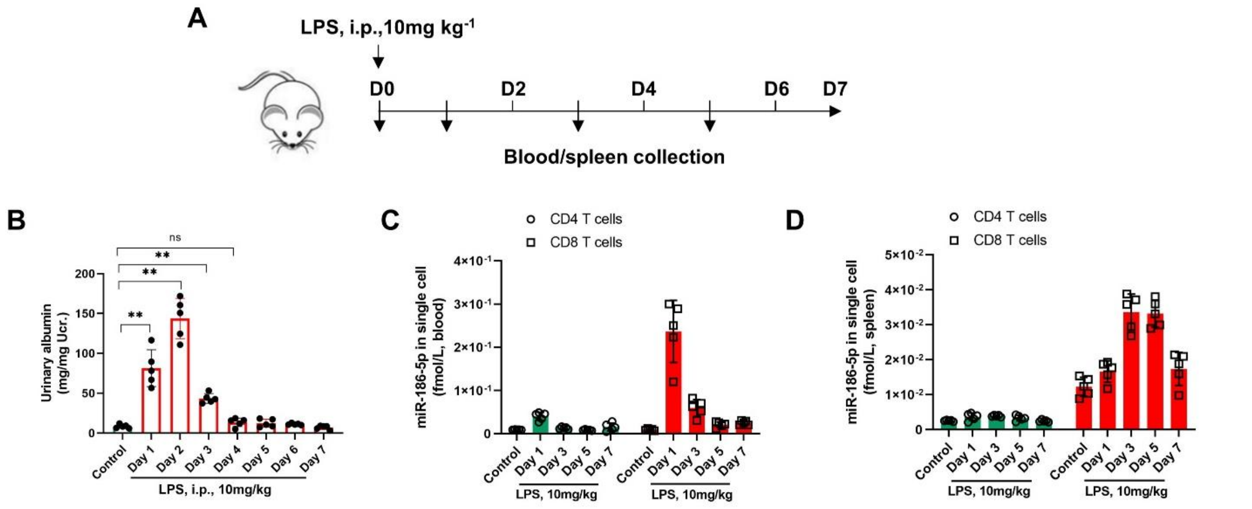

Supplementary Figure 7. LPS-induced mouse T lymphocyte activation model. A) Schematic of mouse T lymphocyte activation model induced by LPS intraperitoneal administration. B) Urinary albumin level in mice with or without LPS treatment. C-D) miR-186-5p levels in CD8 T cells from mouse peripheral blood (C) and spleen (D) with or without LPS treatment. There were 5 mice per group (B-D). Data were analyzed by unpaired two-sided Student's *t* test (B) and presented as group means ± SD. **, $P < 0.01$. ns, no significant.

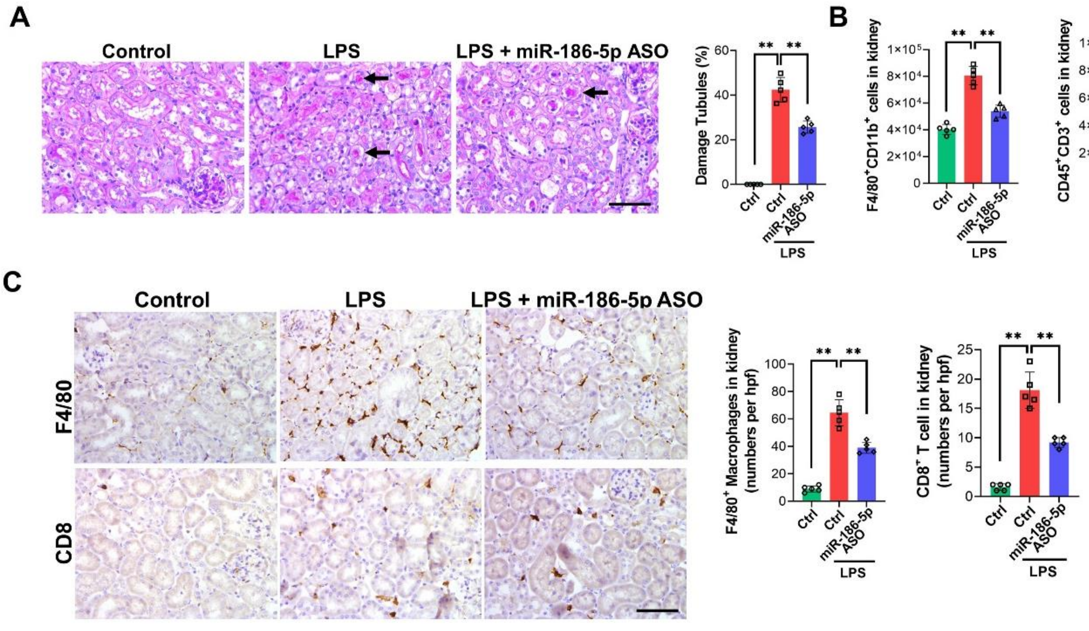

Supplementary Figure 8. Depletion of exosomal miR-186-5p via cholesterol-modified miR-186-5p anti-sense oligonucleotides (ASO) attenuated LPS-induced renal injury and immune cell infiltration. 10 nmol of cholesterol-modified miR-186-5p anti-sense oligonucleotides (ASO) or oligonucleotide control were dissolved in 150μl of PBS with or without LPS and then injected into C57BL/6 mice twice via tail vein. Mice were euthanized 3 days following treatment with or without LPS/ miR-186-5p ASO, and the renal injury and immune cell infiltration were assessed. A) miR-186-5p ASO mitigated LPS-induced renal tubular injury. B-C) miR-186-5p ASO blocked kidney infiltration of macrophages and CD8 T cells assayed by flow cytometry (B) and immunohistochemical staining (C). There were 5 mice per group (A-B); 6-8 fields were analyzed for each mouse (C). Data were analyzed by unpaired two-sided Student's *t* test, and presented as group means ± SD. **, $P < 0.01$. ns, no significant.

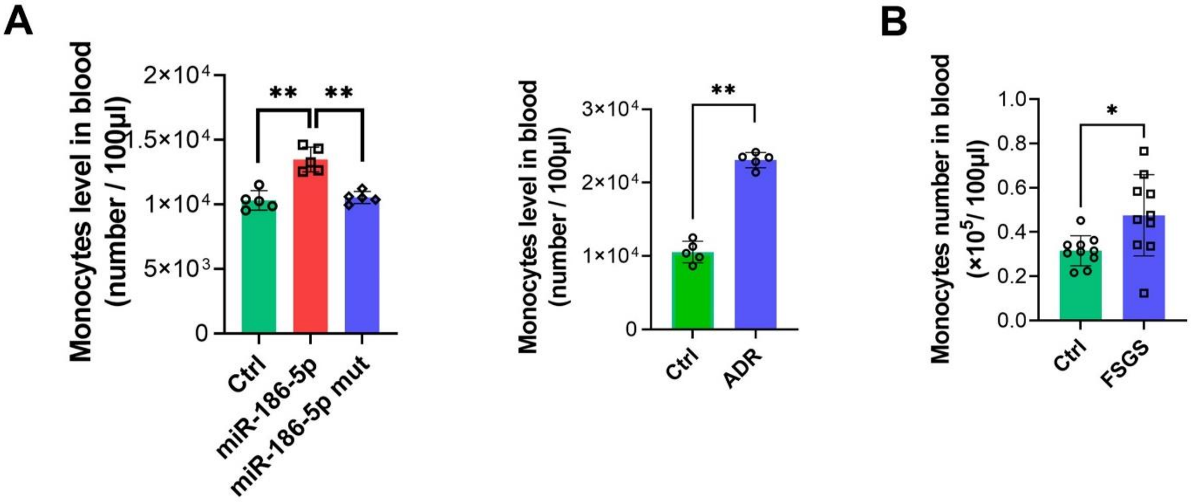

Supplementary Figure 9. Increase of monocyte level in mouse peripheral blood by miR-186-5p or ADR treatment and in FSGS patient peripheral blood. A) Blood monocyte levels in mice injected with miR-186-5p or miR-186-5p mut (left) or treated with or without ADR (right). B) Blood monocyte levels in healthy donors and FSGS patients. There were 5 mice per group (A), 10 healthy control and 10 FSGS patients (B); Data were analyzed by unpaired two-sided Student's t test (A-B) and presented as group means ± SD. *, $P < 0.05$. **, $P < 0.01$.

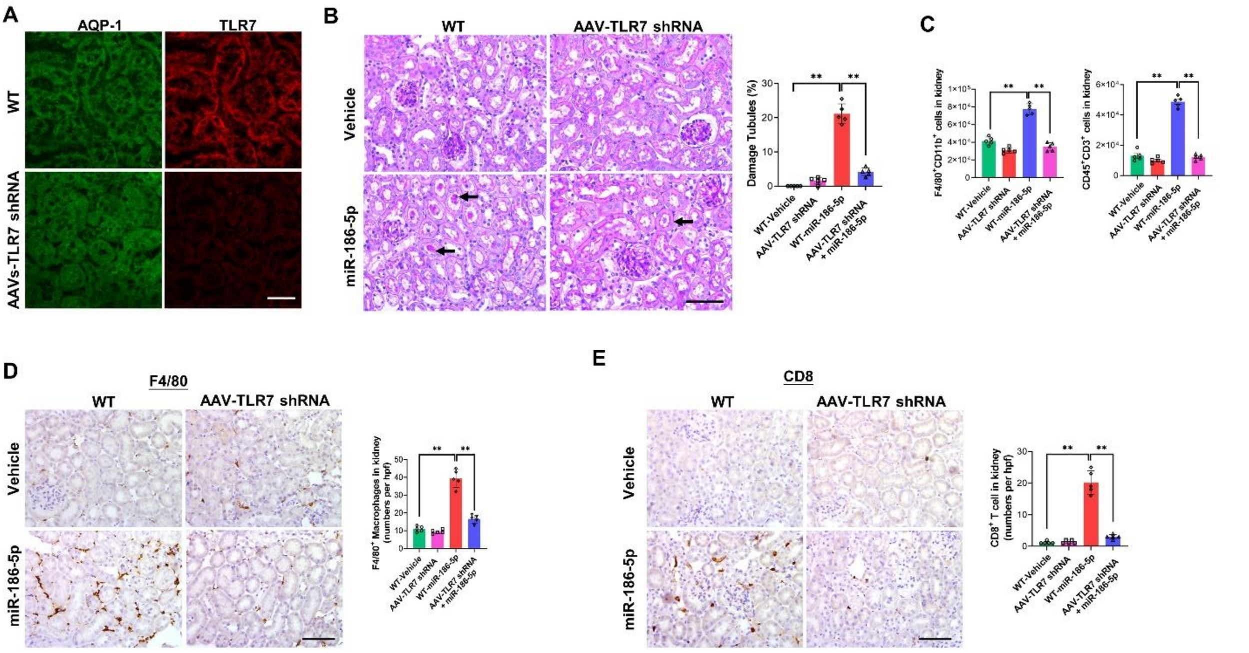

Supplementary Figure 10. Depletion of renal tubular TLR7 via intrarenal injection of AAV-TLR7 shRNA attenuated renal tubular injury and infiltration of immune cells induced by miR-186-5p. TLR7-specific shRNA and scramble shRNA were subcloned into pHBAAV-U6-MCS-CMV-Luc vector, respectively. The vectors were directly injected into the mouse renal cortex. Briefly, 2 weeks prior to ADR treatment, mice were anesthetized by intraperitoneal injections of sodium pentobarbital (30 mg/kg). After the mice were fully anesthetized, the renal was exposed via a flank incision. The adenovirus vectors were slowly injected into the renal cortex at six different sites (10μl solution per each site) with 31-gauge needle. A) Depletion of renal tubular TLR7 by intrarenal injection of AAVs-TLR7 shRNA. B) Renal tubular TLR7 reduction attenuated kidney injury induced by miR-186-5p. C-E) Renal tubular TLR7 reduction inhibited kidney infiltration of F4/80^{+}CD11b^{+} macrophages and CD45^{+}CD3^{+} or CD8 T cells induced by miR-186-5p assayed by flow cytometry (C) and immunohistochemical staining (D, E). There were 5 mice per group (A-C); 6-8 fields were analyzed for each mouse (D-E). Data were analyzed by unpaired two-sided Student's *t* test, and presented as group means ± SD. **, $P < 0.01$. ns, no significant.

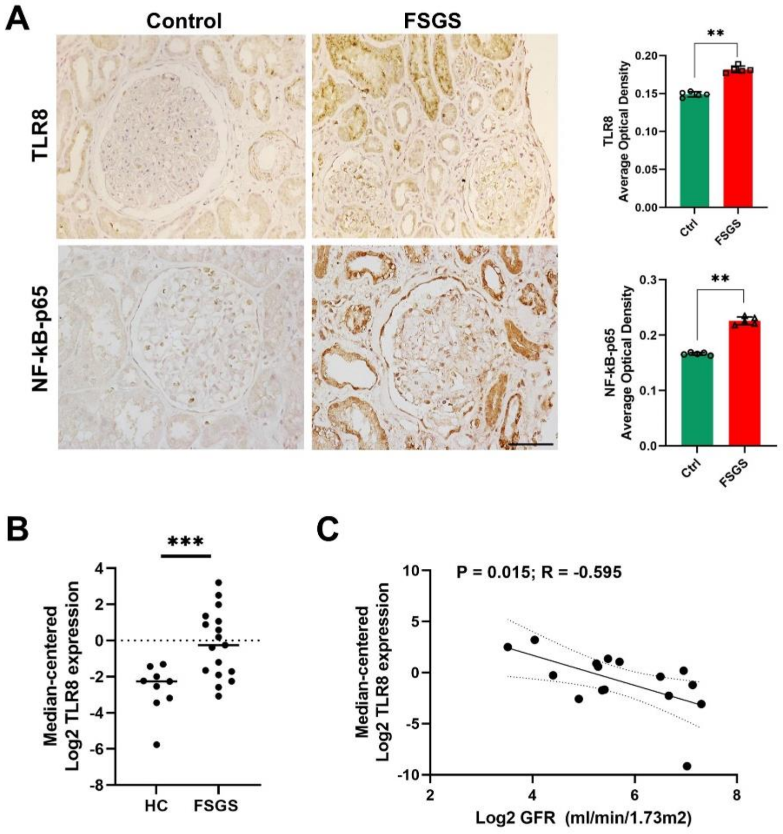

Supplementary Figure 11. Potential role of TLR8 signal in renal inflammation in FSGS patients. A) Enhanced TLR8 and NF-κB-p65 expression in renal tubules of FSGS patients. B) Renal tubular TLR8 level in FSGS patients and control donors (HC). C) The correlation between TLR8 level and GFR in FSGS patients. Panel B and C were derived from analysis of the NephroSeq online database (GSE108112). Scale bars, 50μm. n = 5 patients or control (A), 6-8 fields were analyzed for each patient or control (A). Data were analyzed by unpaired two-sided Student's t test (A, B) or Spearman's correlation test, and presented as group means ± SD. **, $P < 0.01$. ***, $P < 0.001$.

Raw WB data

Figure 3D

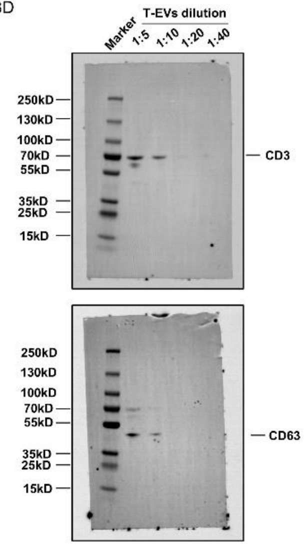

Figure 6G

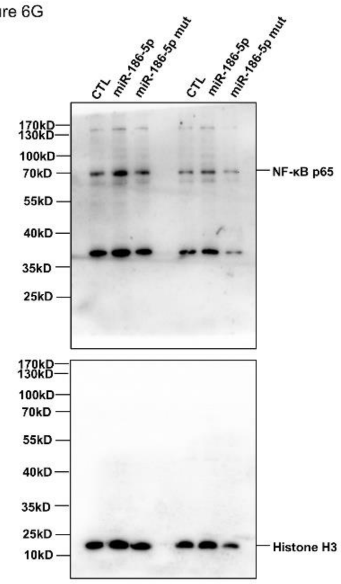

Figure 7A

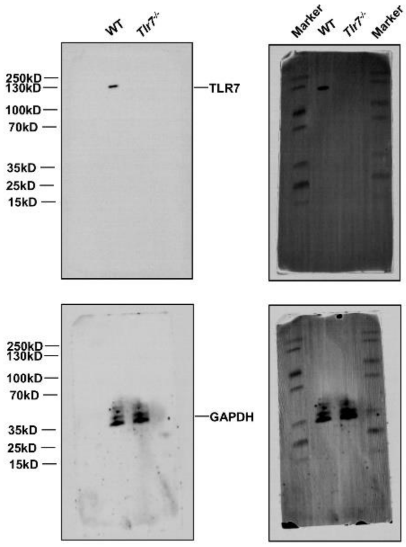

Supplement: Supplementary file 1 — Supporting Information [file ADVS-10-2301492-s001.pdf]
